# Host-Associated Bacterial Succession during the Early Embryonic Stages and First Feeding in Farmed Gilthead Sea Bream (*Sparus aurata*)

**DOI:** 10.3390/genes10070483

**Published:** 2019-06-26

**Authors:** Eleni Nikouli, Alexandra Meziti, Efthimia Antonopoulou, Eleni Mente, Konstantinos Ar. Kormas

**Affiliations:** 1 Department of Ichthyology and Aquatic Environment, School of Agricultural Sciences, University of Thessaly, 384 46 Volos, Greece; 2 Laboratory of Animal Physiology, Department of Zoology, School of Biology, Aristotle University of Thessaloniki, 541 24 Thessaloniki, Greece

**Keywords:** sea bream, development, larvae, symbionts, bacteria

## Abstract

One of the most widely reared fish in the Mediterranean Sea is *Sparus aurata*. The succession of *S. aurata* whole-body microbiota in fertilized eggs, five, 15, 21 and 71 days post hatch (dph) larvae and the contribution of the rearing water and the provided feed (rotifers, *Artemia* sp. and commercial diet) to the host’s microbiota was investigated by 454 pyrosequencing of the 16S rRNA gene diversity. In total, 1917 bacterial operational taxonomic units (OTUs) were found in all samples. On average, between 93 ± 2.1 and 366 ± 9.2 bacterial OTUs per sample were found, with most of them belonging to Proteobacteria and Bacteroidetes. Ten OTUs were shared between all *S. aurata* stages and were also detected in the rearing water or diet. The highest OTU richness occurred at the egg stage and the lowest at the yolk sac stage (5 dph). The rearing water and diet microbial communities contributed in *S. aurata* microbiota without overlaps in their microbial composition and structure. The commercial diet showed higher contribution to the *S. aurata* microbiota than the rearing water. After stage D71 the observed microbiota showed similarities with that of adult *S. aurata* as indicated by the increased number of OTUs associated with γ-Proteobacteria and Firmicutes.

## 1. Introduction

To date, it is widely accepted that the gastrointestinal tract in humans and other animals contain complex communities of microbial populations involved not only in the digestion and nutrition but also in the overall health of the host [1]. Early studies suggested that the gastrointestinal tract of a human fetus is sterile, and that microbial colonization starts soon after birth [2]. Additional evidence supports that birth delivery type, i.e., cesarean versus vaginal, also affects the initial structure of the infant gastrointestinal microbiome [3,4]. However, recent studies indicate that maternal transition of internal microbes also takes place and shapes fetal fecal microbiota [5,6,7]. Priority effects, i.e., how the magnitude and timing of arrival of gut microbes in early life stages impacts the host’s health in the long term [8] along with the gut microbial succession and functional maturation in infants seem to be connected with the dietary changes during the early life (breast/formula milk/solid foods). Initial microbial colonization plays a crucial role on gastrointestinal development and also affects the health of the host in later life [9]. Similarly, the presence of microorganisms in aquatic animals has been well documented through previous studies, with bacterial species being beneficial in nutrition and their overall health [10].

In fish hatcheries where the rearing conditions are considered more controlled in contrast to the open sea cages environment, researchers have shown that fertilized eggs are colonized with microbial populations in abbreviated time, that then colonize the growing embryo and subsequently the fish larvae [11]. The presence of microbial communities has also been documented in fish eggs in nature. However, in intensive rearing, eggs hatch in higher densities than in nature, while their microbial communities also differ in abundances and structure [12]. It has also been reported that the newly hatched fish larvae consume egg fragments together with their microbial communities [13,14,15]. This, along with the rearing water and live feed microbiota, can crucially affect, the gut bacterial community structure in fish species [16]. However, recent studies indicate that there is also a strong relation between gut microbial succession and host development [17,18]; While Dehler et al. [19] have shown that host physiology also affects gut microbial colonization in Atlantic salmon (*Salmo salar*) parr, Vadstein et al. [20] reported that the larvae microbiota changes fast until metamorphosis, due to changes in host-microbe and microbe-microbe interactions.

Findings like these suggest that host-microbiota interactions can be used in aquaculture for selective microbial manipulation aiming to promote beneficial symbiosis [21]. Despite the fact that *S. aurata* is a high value farmed fish species for many European countries, research on its standard gut microbiota has been focused in adult individuals [22,23,24,25] and also in the evaluation of how it is affected by consuming formulated diets containing alternative ingredients [26,27,28,29,30,31,32]. On the other hand, only a few studies exist about the microbial colonization in healthy populations of *S. aurata* larvae during early life based both on cultured methods [33,34] and next generation sequencing [35]. All of them highlight the significance of live preys’ microbiota in intestinal microbial communities of *S. aurata* during the first feeding. Despite that the findings by Califano et al. [35] uncover the bacterial profiles in *S. aurata*, along with its rearing water and feeds, they investigated only the initial associated bacterial community (on two days after hatching) and how it was shaped 32 days later, without examining developmental or trophic stage specific effects on the structure and composition of bacterial communities in the selected microhabitats. Additionally, none of the above studies have taken into consideration the structure and fate of the microbial populations of the fertilized eggs and whether the microbiota contribute to the initial bacterial establishment in the larval gut. More than that, there is no assessment on bacterial communities during the transition from the live feed to artificial diet.

The aim of this study was to investigate the host-associated microbial succession in farmed *S. aurata*, from fertilized egg up to 71 days post hatch (dph) larvae. In addition, the contribution of the rearing environment and supplied diet to host-associated bacterial communities was examined by performing intermediate samplings just before the change of each trophic stage (yolk sac absorption, live feeds, commercial diet). Moreover, the analysis of the rearing water and diet microbiota was performed. The experiment was conducted in a commercial hatchery facility in order to get results that reflect the microbial niches that occur under contemporary commercial farming conditions. 

## 2. Materials and Methods 

### 2.1. Rearing Conditions

The experiment was conducted under commercial farming conditions in 72 days period (between November 2015 and January 2016) in a Greek aquaculture commercial unit. Fertilized eggs were derived from the same pair of spawners. Breeding and hatching were carried out in cylindrical tanks with a flow through system using natural sea water (treated with mechanical filtration and UV light sterilization) according to the guidelines of Moretti et al. [36,37]. Physical and chemical parameters (pH, salinity, temperature, dissolved oxygen, nitrate, phosphate) were monitored daily; throughout the experiment these variables remained stable and within the optima values used in *S. aurata* hatcheries (data not shown). Hatching and the yolk sac stage were carried out in static water, while growth in the rest of the trophic stages there was performed in a continuous water flow-through.

### 2.2. Sampling

**Figure 1:**
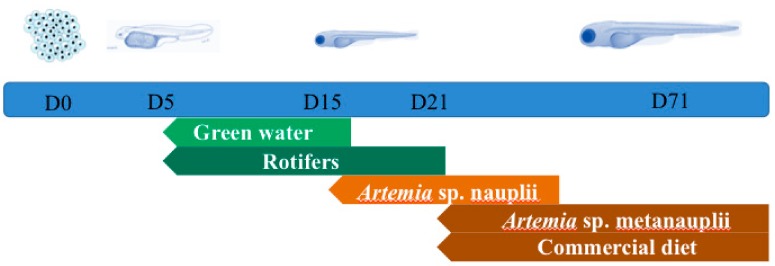
Experimental design and trophic stages from day zero (D0) to day 71 (D71).

Samples were collected at the end of each of the five different time points (Figure 1) representing developmental stages of *S. aurata* when the feeding type changes. Gut tissues were not dissected out due to the small size of the larvae and for this we analysed whole-body microbiota. On each sampling point (*n * = 5) three individual samples consisting of ~0.25 g of pooled samples of eggs (day zero) or larvae (D5, D15, D21, D71; days post hatching) were collected aseptically and anaesthetised with MS222. Similarly, pooled triplicates were taken also from the feeds: Rotifers (RT), *Artemia salina* nauplii (AN), *Artemia salina* metanauplii (AM), and commercial diet (CD). After each sampling, all the samples (except CD) were thoroughly rinsed under rigorous mechanic agitation three times consecutively with sterile particle free seawater, to reduce skin and the surrounding environment associated bacteria. Furthermore, in each time point, 2 L of water were collected from the rearing tank in sterile flasks, and were immediately vacuum filtered (<150 mm Hg) through a 0.2 μm filter (GTTP, Millipore, MA, USA). All the samples were stored at −80 °C until further analysis.

### 2.3. DNA Isolation and Sequencing

For the identification of the larvae and feed microbiota, three individual biological replicates of ~0.25 gr of pools from each sample type (*n* = 5) from each time point (*n* = 5) were used. DNA was extracted using the QIAGEN QIAamp DNA Mini Kit (Qiagen, Hilden, Germany) following the manufacturer’s protocol “DNA Purification from Tissues”. For the water samples, the PowerSoil DNA Isolation kit (MoBio, Carlsbad, CA, USA) was used according to the manufacturer’s protocol as this kit is more widely used for water samples and also allowed us direct comparisons with our previously analysed water samples (data not shown). Prior to extraction, each filter (*n* = 5) was cut in three equal pieces and each piece was used as an individual replicate for each time point. 

We analyzed the 16S rRNA gene bacterial diversity from each individual sample, targeting the V3-V4 region by using 454 pyrosequencing with the primer pair S-D-Bact-0341-b-S-17 and S-D-Bact-0785-a-A-21 [38]. Samples were sequenced utilizing Roche 454 FLX titanium instruments and reagents after following the manufacturer’s guidelines at the MRDNA Ltd. (Shallowater, TX, USA) sequencing facilities [39]. Pyrosequencing reads were processed by the MOTHUR platform (version 1.38) [40,41] following the same walk-though described previously in Nikouli et al. [25]. The operational taxonomic units (OTUs) were classified by the SILVA Incremental Aligner (SINA) online alignment service for small (16S) subunit ribosomal RNA [42], by setting minimum identity with query sequence 0.95 and by rejecting sequences below identity 80%. The sequences that could not be classified into any known phylum were assigned as “unclassified” from the SILVA database, release 130 [43]. Raw sequence data from this study have been submitted to the Sequence Read Archive (https://www.ncbi.nlm.nih.gov/sra/) with accession number PRJNA494043. Statistical analysis and graphical illustrations were performed using the Palaeontological Studies (PAST) software [44] and the R Studio platform [45].

## 3. Results

**Figure 2:**
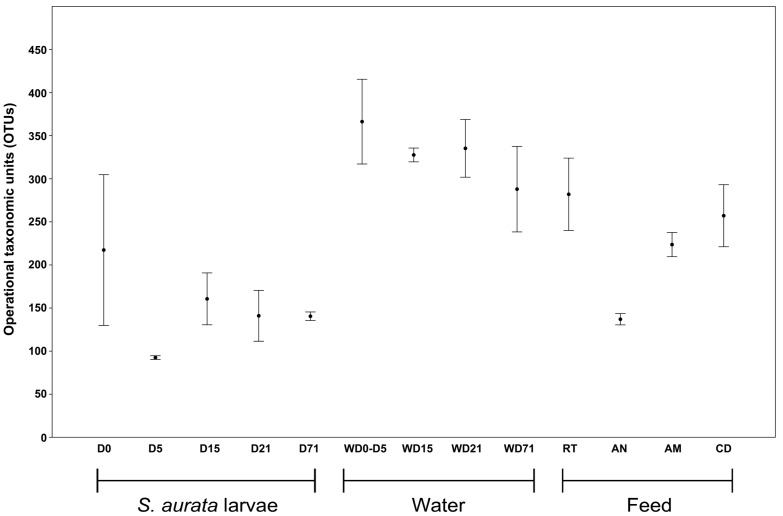
Boxplot of observed species richness in *Sparus aurata* larvae, water and feed samples bacterial communities. D: Day, AM: *Artemia salina* metanauplii, AN: *Artemia salina* nauplii, CD: Commercial diet, RT: Rotifers, W: Water sample.

A total of 1917 operational taxonomic units (OTUs) remained in the dataset after the quality filter and the removal of single singletons (Table S1). Based on the rarefaction curves (Figure S1) and the Chao1 index (Table 1), sequencing depth was satisfactory for the majority of the samples. The comparison of rarefied and non-rarefied data showed that although, as expected, the number of OTUs decreased in the rarefied dataset, the coverage was ≥ 90% (Table S2). The average number of OTUs per sample (Table 1) ranged from 93 ± 2.1 (D5) to 366 ± 49.2 (WD0_5). The lowest OTUs richness occurred at D5 (93 ± 2.1) and the highest at D0 (217 ± 87.5). Statistically significant different OTUs richness (ANOVA F = 7.98, *p <* 0.001) (Table 2) in the developmental stages of *S. aurata* occurred in stage D5, which was different with stages D15, D21 and D71 (Table 2). OTUs richness did not change significantly in the rest of the time points after the mouth opening (D15, D21, D71). The number of species in the rearing water and diet (with only exception the AN samples) were always higher than the species richness in *S. aurata* in every time point (Figure 2). Bacterial diversity assessed by the Shannon H and Simpson 1-D indices, suggested that low diversity communities occurred in all sample types, which were dominated by only a few species (Table 1).

**Figure 3:**
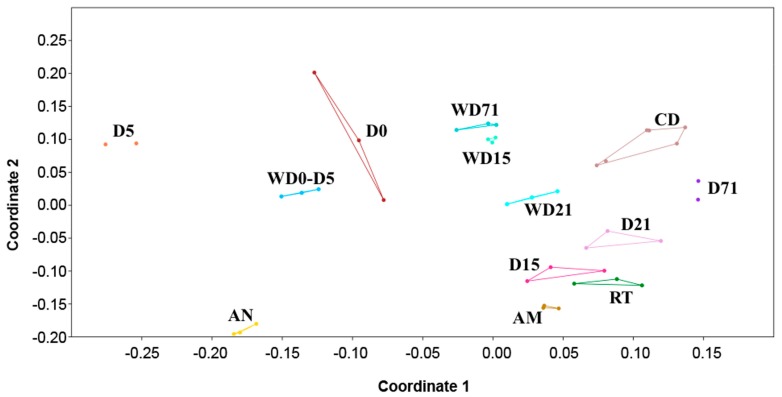
Non-metric multidimensional scaling (nMDS) plot displays of the bacterial communities of all sample categories (larvae, rearing water and feeds) based on Bray–Curtis distances. D: Day, AM: *Artemia salina* metanauplii, AN: *Artemia salina* nauplii, CD: Commercial diet, RT: Rotifers, W: Water sample.

**Figure 4:**
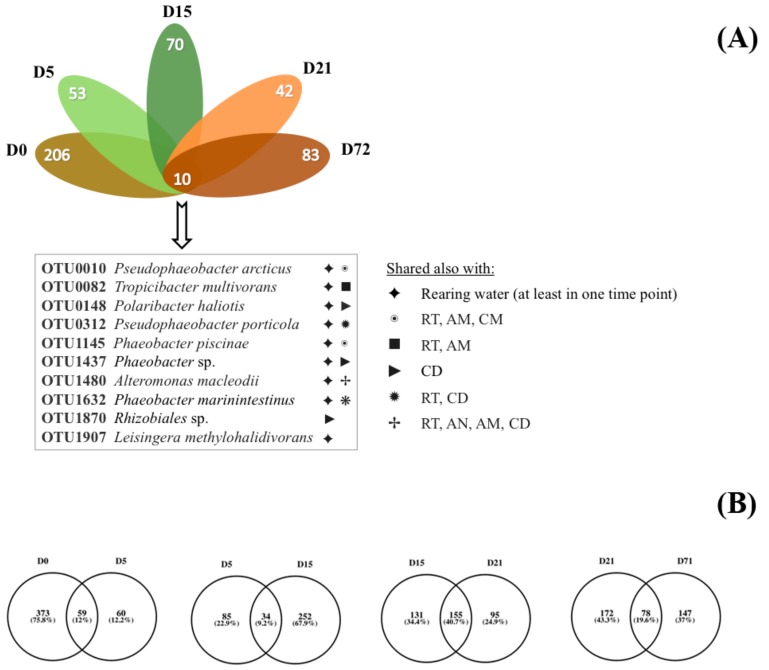
Flower diagram (**A**) of the shared and unique (**B**) operational taxonomic units (OTU) in *Sparus aurata* larvae from day (D) zero to day 71. AM: *Artemia salina* metanauplii, AN: *Artemia salina* nauplii, CD: Commercial diet, RT: Rotifers.

Nonmetric multidimensional scaling (NMDS) based on the Bray–Curtis dissimilarity distance matrix was used for comparing the bacterial communities in all sample categories from all time points, and it showed no overlap and clear separation between different sample types (Figure 3). The clustering of NMDS was also supported by both weighted and unweighted UPGMA (unweighted pair group method with arithmetic mean) clustering (Figure S2). By comparing the bacterial communities between the samples, it was found that 10 OTUs were shared between the *S. aurata* larvae samples in all five time points (Figure 4A, Figure S3), which are shared with diets or rearing water. The closest taxonomical relatives of the shared OTUs across all samples were *Pseudophaeobacter arcticus*, *Tropicibacter multivorans, Polaribacter haliotis, Pseudophaeobacter porticola, Phaeobacter piscinae, Phaeobacter* sp., *Alteromonas macleodii, Phaeobacter marinintestinus,* Rhizobiales, and *Leisingera methylohalidivorans*. The highest percentage of shared OTUs between two consecutive stages occurred during the transition from D15 to D21 (40.7%) and the lowest (9.2%) from D5 to D15 (Figure 4B). However, in every time point 19.2%–60.9% of OTUs were found to be unique only in the host samples, with D0 having the highest occurrence of unique OTUs (60.9%). After the mouth opening, a reduction in *S. aurata* larvae stage specific OTUs was observed, while more shared OTUs were found in with the feeds, rather than those from the rearing water (Figure 3, Figure S3).

**Figure 5:**
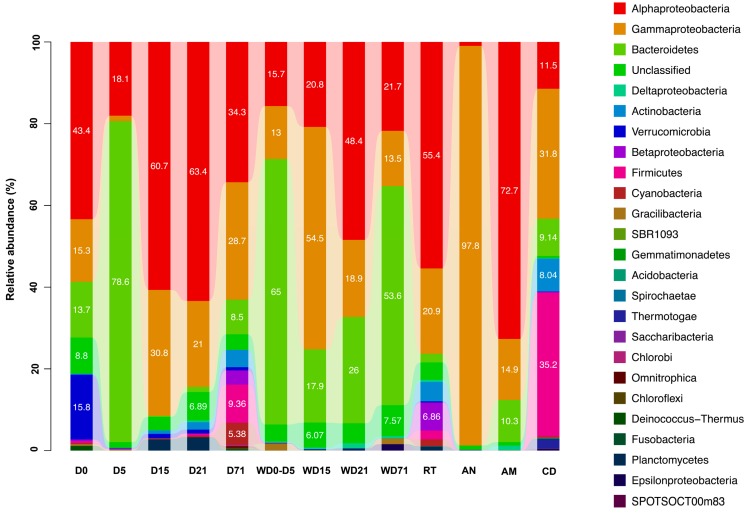
Relative abundance of bacterial phyla and Proteobacterial classes in all sample categories (*Sparus aurata* larvae, rearing water and feeds) D: day, AM: *Artemia salina* metanauplii, AN: *Artemia salina* nauplii, CD: commercial diet, RT: rotifers, W: water sample.

From the phylogenetic analysis 19 bacterial phyla (Figure 5) were found (Proteobacteria, Bacteroidetes, Firmicutes, Verrucomicrobia, Actinobacteria, Cyanobacteria, Planctomycetes, Fusobacteria, Gracilibacteria, Deinococcus-Thermus, Chloroflexi, Omnitrophica, Chlorobi, Saccharibacteria, Thermotogae, Spirochaetae, Acidobacteria, Gemmatimonadetes and SBR1093) with predominance of Proteobacteria and Bacteroidetes (65.0% and 22.0% respectively of the total relative abundance). The rest of the found taxa were present with relative abundance <1.0%, except the phylum Actinobacteria (1.6% of the relative abundance). A total of 4.1% of the sequences could not be assigned to any of the known bacterial phyla and were designated as “unclassified”.

Bacterial community profiling in *S. aurata* samples revealed shifts in the structure and composition between the five trophic stages examined here (D0, D5, D15, D21, D71) (Figure 5, Figure S4). More specifically, Proteobacteria dominated in D0 (59.2% relative abundance) followed by Bacteroidetes with low relative abundance (13.7%). However, the proportion of Proteobacteria decreased from D0 to D5, giving way to Bacteroidetes (78.6%), and increased again at D15. From D15 to the end of the trial the contribution of Bacteroidetes in the microbiome of *S. aurata* was relatively low, with Proteobacteria dominating again with relative abundance 66.5%–91.6%. The predominance of Bacteroidetes in D5 was due only to *Tenacibaculum* representatives (77.0% relative abundance). This genus was found among the Bacteroidetes of the rearing water samples with 0.04% (D15)–10.57% (D0–D5) relative abundance. Within the Proteobacteria (Figure S4), α-Proteobacteria class dominated (with 18.1%–63.4% relative abundance), followed always by γ-Proteobacteria. The family Rhodobacteraceae was the most abundant in all time points (25.2%–60.3% relative abundance), except D5 when Proteobacteria was the second most abundant phylum due to the OCS116 family (16.7% relative abundance). This family contributed for 10.1% relative abundance in D0, while Pseudoalteromonadaceae in D15 (19.9%), Vibrionaceae in D21 (13.5%) and Moraxellaceae in D71 (9.6%).

The composition of the bacterial diversity in the rearing water also changed between the sampling periods. Initially, when the rearing water was static (WD0–WD5), bacterial communities were dominated by Bacteroidetes (65.0% relative abundance). In the time points WD15–WD21, Proteobacteria was the most abundant phylum (with relative abundance 75.7% and 68.6% respectively), until D71 when Bacteroidetes were found again in high proportion (53.6% relative abundance). Proteobacteria in WD0_5, WD21, and WD71 were mainly represented by the Rhodobacteraceae (14.4%, 45.8% and 21.4% relative abundances, respectively) and Oceanospirillaceae (in WD15 with 51.1% relative abundance) families. Bacteroidetes were mainly represented by the Flavobacteriaceae family, which increased in relative abundance across the experiment (from 12.6% relative abundance in WD0_5 to 37.9% in WD71). However, in water WD0_5, Cryomorphaceae are the dominant family (51.9% of the relative abundance).

Live feed samples, i.e., rotifers and *A. salina* nauplii and metanauplii, consisted almost exclusively of Proteobacteria with relative abundance 83.7%, 98.8% and 88.8%, respectively, although the representatives of this phylum differed between the three live feeds. Rotifers were represented mainly by α-Proteobacteria (55.5% of the relative abundance) and, in particular by the Rhodobacteraceae (31.7% relative abundance) and Phyllobacteriaceae (20.2% relative abundance) families. The γ-Proteobacteria class showed 20.9% relative abundance, consisting mostly of species affiliated to the Vibrionaceae (6.7% relative abundance), Enterobacteriaceae (3.7% relative abundance) and Oceanospirillaceae (2.9% relative abundance) families. On the other hand, *Artemia* sp. nauplii consisted almost entirely of γ-Proteobacteria and more specifically of members of the Alteromonadaceae family (85.9% relative abundance), while *Artemia* sp. metanauplii showed dominance by species belonging to the α-Proteobacteria family Rhodobacteraceae (70.1% relative abundance). Commercial diets OTUs were related to the Proteobacteria, Firmicutes and Bacteroidetes phyla, with 44.0%, 35.2% and 9.1% relative abundance, respectively. Within the Firmicutes, the retrieved OTUs belonged to the following families: Streptococcaceae (8.9%), Staphylococcaceae (6.7%), Lactobacillaceae (6.7%), Bacillaceae (1.8%) and Clostridiaceae (7.3%). The Proteobacteria mainly consisted of γ- (31.8%) and α-Proteoabcteria (11.5%) with representatives from Vibrionaceae (17.5%), Rhodobacteraceae (10.5%) and Oceanospirillaceae (9.0%) families. 

## 4. Discussion

One of the frequent areas of research in animal-host interactions is the change of the animal’s microbiome along its ontogeny. The structure of microbial communities can be drastically altered along the developmental changes in many animals [46] and humans [47]. In this study the microbiota succession in whole body larvae of *Sparus aurata* from the egg to 71 dph was assessed in relation to the microbiota of the rearing environment and supplied diet. The associated bacterial community in early trophic stages in healthy populations of *S. aurata* and the effect of rearing conditions on their gastrointestinal microbial structure and composition, have only partially been studied [33,34,35]. Califano et al. [35] used for the first time next generation sequencing (NGS) tools in order to uncover in high-resolution the diverse microbial communities in a *S. aurata* larviculture system. However, they studied only the initial microbial diversity (on samples from 2 dph) and its shaping 32 days later. The studied parameters were rearing water and live feeds without further investigation on the bacterial community shifts after the inclusion of artificial formulated commercial diet in the next stages or any contribution of the egg’s microbiota. The most important difference between the present work and the Califano et al. [35] study, is the examination of the trophic stage-specific effect on the structure and composition of the host-associated bacterial communities in *S. aurata*. This was achieved by performing additional intermediate samplings during the rearing period, every time before the diet was changed in order to capture stage-specific microbial communities between the different sample types. However, their findings are in agreement with the findings of this study that live feed and the surrounding rearing environment contribute to the microbiome of sea bream larvae differently depending on the stage of the larval development.

Additionally, the present study profiled for the first time the bacterial community in healthy *S. aurata* eggs and recorded a high proportion (34.5%, Figure S5) of bacterial representatives that are not shared with the microbial community of the rearing water (D0). Possibly these bacteria originated from the water in the spawning tank. However, Hansen and Olafsen [48], recorded the presence of microbial populations in eggs inside the ovary in healthy individuals of cod (which had been removed under aseptic conditions). This finding also supports the hypothesis that this unique bacterial community that has been found exclusively in eggs is part of their autochthonous microbial community. The present study, however, cannot rule out that part of the egg’s microbiota could be originated from rare bacteria of the seawater that were not detected in the samples analysed. As the bacterial communities in the water column have, in general, higher species richness than the ones in the gut, and since the two kits might have differential DNA extraction potential for the gut tissue and the water samples, part of the observed differences in the resulting sequence reads could be attributed to the two kits used. For these reasons, more experimental investigation of the origin of egg microbiota is required in the future.

At the yolk sac stage (D5) the host associated OTUs showed high similarity (77.3%, Figure 3) with those of the rearing water. These bacteria are probably coming from the surrounding water as a result of osmoregulation processes taking place before the full absorption of the yolk sac [48,49]. Stage D5 also reported a significant lower species richness compared to larvae samples in the other time points reported both from the observed and estimated richness (Chao1). At D15 (after mouth opening and live feed addition), bacterial species richness increased 1.7 times. This relation between the first feeding and bacterial species richness has been also reported in other fish species [50,51,52]. Savaş et al. [34] reported also an increase in *S. aurata* associated bacterial communities after the yolk sac stage, but they studied only the aerobic populations.

From D15 and later, a gradual decrease was found in the number of shared OTUs between the rearing water and larvae, while the provided feed had higher contribution in the *S. aurata* bacterial richness. This is opposed to the findings by Bakke et al. [53] who reported higher impact of the rearing water in the cod (*Gadus morhua*) larvae microbiota fed with live feed but this remark should be taken cautiously as Bakke et al. [53] used a different DNA extraction protocol and bacterial diversity was assessed with denaturing gradient gel electrophoresis (DGGE) which restricts direct comparison of the two studies. Furthermore, 14 OTUs were shared in *S. aurata* samples at D0 and D15, that have not been previously detected in D5, supporting the concept that fish larvae during the first feeding can consume egg fragments and, thus, part of their microbiota [13,14,15]. According to Bates et al. [54], these bacterial communities play an important role in the formation and function of the gastrointestinal tract in fish species. The diets used in this study were characterized by high bacterial species richness. Rotifers have been found to carry large numbers of bacteria per individual [55,56,57,58] that may be due to their gastrointestinal tract microbiota, as they ingest bacteria from their rearing environment [59]. Low bacterial diversity in the AN sample, is probably due to the fact that *Artemia* sp. cysts are sterilized by immersion in chlorine solutions prior to their decapsulation. Soon after hatching, they are distributed directly to the rearing fish tanks with this time span not being long enough to “build” a more complex bacterial community. In order to produce AM, nauplii are bred for about 12 h with commercial nutrient enrichment [36], which could enhance species richness increase. 

Bacterial diversity in the rearing water samples was always higher compared to the rest of the samples in each time point. This finding is in agreement with previous studies in similar rearing systems [35,60]. The occurrence of shared OTUs between the *S. aurata* larvae and the water or the provided feed microbiota is restricted to a few OTUs and there is no community overlap between them. The bacterial composition in the *S. aurata* samples consisted mostly of Proteobacteria and Bacteroidetes, two characteristic phyla of the gastrointestinal bacterial community in many marine fish species [23,28,61,62,63]. It is interesting that in *S. aurata* early life stages, the proteobacterial OTUs (especially at D0, D5, D15, D21) belonged mainly to the α-Proteobacteria, while this class has been found in low relative abundances in adult individuals. Moreover, Firmicutes largely contribute in the gut microbiota of *S. aurata* adult individuals, but their proportion remained low in all life stages studied here. However, at D71 a microbiota transition occurred with an increase in γ-Proteobacteria and Firmicutes proportion, that could be attributed to the transition to commercial diet and seems to be evidence of early maturation of the host-associated bacterial community in *S. aurata* [28].

Bacteroidetes, was the dominant phylum only in D5, represented mainly by the *Tenacibaculum* species. This genus consists of species of marine origin but also fish pathogens [64], like *Tenacibaculum ovolyticum* that can dissolve the chorion of Atlantic halibut (*Hippoglossus hippoglossus*) eggs, damage the zona radiata and lead to larval death [65]. The dominant OTU at D5 (Table 1) is closely related to *Tenacibaculum dicentrarchi*, that has been previously found in diseased European sea bass (*Dicentrarchus labrax*) [66] and *Salmo salar* individuals [67,68]. Despite these previous findings, there were no indications of pathogenicity in eggs or in larvae through the experiment presented here.

At the D0, D15, D21, D71 time points the dominant OTUs were affiliated with bacterial species of fecal/gut origin isolated from fish and other aquatic animals, with some of them known to be beneficial to their hosts [69,70,71]. For the developmental stages of *S. aurata* specifically investigated in this study, it remains to be explored whether these bacteria have such roles. A first step towards this direction for adult *S. aurata* has been reported in Nikouli et al. [25] where the potentially fast-growing gut bacteria were recognized as a first criterion for their proliferation in the fish gut habitat. In D15 and D21 when larvae were fed with live feeds, the dominant OTU is associated with the anaerobic species *Ruegeria mobilis*, which possess both planktonic lifestyle and biofilm formation capabilities, optimum growth in pH 7 and low NaCl concentrations [72], containing genes that enhance antibiotic tropodithietic acid (TDA) production [73]. Tropodithietic acid from other bacterial species has been found to reduce *Vibrio anguillarum* in cod larviculture [74]. All these characteristics constitute a probiotic profile for OTU0006 that has also been found in lower proportion in D0 (0.29% relative abundance) and enhancing, thus, the hypothesis for possible vertical transmission. Finally, the dominant OTU at D71 (OTU0025) is related with the bacterial species *Photobacterium phosphoreum*, which is associated with spoilage of fish products but also have been found in the normal gut microbiota of other fish species [75,76] with unknown role in the gastrointestinal ecosystem. However, the high abundance of this species at D71 is maybe due to the high proportion that this OTU also had at the commercial diet (that *S. aurata* larvae were feed at D71). 

## 5. Conclusions

The present study profiled the bacterial communities associated with the early life and trophic stages of *S. aurata* and the effect of the surrounding environment and diet in early bacterial colonization and succession. Results presented here, reported stage specific microbial enrichments and also shifts in the contribution of the rearing environment to host-associated bacterial communities. Along the five investigated developmental stages, our study revealed the presence of common bacterial species in the host between the five ontogenetic/trophic stages, that have also been identified in the rearing water or in diet samples. However, unique bacterial OTUs in the host (that are not in common with the rearing environment) were detected in all time points. Such stage-specific bacteria could have important functional roles associated with the ontogeny of *S. aurata* and their metabolic potential needs to be specifically investigated. Comparing the microbiome of larvae (D0 to 71) with the bacterial communities of the surrounding rearing environment (water and diet samples), revealed no strong relation between water and the host in any of the five life stages. These findings suggest that microbial communities of the rearing water and diet do not participate directly in shaping the *S. aurata* microbiota, as they have little overlaps between their microbial composition and structure. However, the contribution of the detected bacterial species to the host were unclear and future research should focus on evaluating the functions of these species in order to understand their role in *S. aurata* overall performance.

## Figures and Tables

**Table 1:** Pyrosequencing results of 16S rRNA gene diversity reported in each sample group. N: Number of biological replicates analyzed, D: Day, OTUs: Operational taxonomic units.

Sample	Code	Reads	Observed OTUs Richness	Chao1	Simpson 1-D	Shannon H	No. of the Most Dominant OTUs (Cumulative Relative Dominance ≥ 80%)	Most Abundant OTU, Dominance (%) and Closest Relative (≥97%)
*Sparus aurata*	D0	4525 ± 1930.9 *Ν =* 3	217 ± 87.5	260 ± 104.4	0.89 ± 0.082	3.46 ± 0.714	53 (80.1)	OTU0026 (13.4) *Rubritalea* sp.
	D5	6873 ± 1065.6 *Ν =* 2	93 ± 2.1	125 ± 23.7	0.82 ± 0.031	2.40 ± 0.049	7 (80.3)	OUT0007 (29.0) *Tenacibaculum dicentrarchi*
	D15	2831 ± 1521.6 *Ν =* 3	161 ± 30.1	229 ± 12.6	0.89 ± 0.041	3.34 ± 0.340	30 (80.3)	OUT0006 (28.4) *Ruegeria mobilis*
	D21	1064 ± 447.7 *Ν =* 3	141 ± 29.5	218 ± 16.1	0.93 ± 0.031	3.68 ± 0.376	45 (80.0)	OTU0006 (16.0) *Ruegeria mobilis*
	D71	1347 ± 494.3 *Ν =* 2	141 ± 4.9	197 ± 14.4	0.97 ± 0.001	4.19 ± 0.035	68 (80.2)	OTU0025 (6.1) *Photobacterium phosphoreum*
Rearing water	WD0_5	11,331 ± 4103.6 *Ν =* 3	366 ± 49.2	436 ± 51.5	0.91 ± 0.006	3.73 ± 0.075	39 (80.1)	OTU0002 (27.4) clone Woods-Hole_a1725
	WD15	10,261 ± 1145.7 *Ν =* 3	328 ± 8.0	411 ± 19.8	0.85 ± 0.014	3.34 ± 0.085	29 (80.2)	OTU0001 (36.1) *Marinobacterium marisflavi*
	WD21	4634 ± 2064.9 *Ν =* 3	335 ± 33.5	440 ± 46.5	0.94 ± 0.008	4.02 ± 0.052	57 (80.1)	OTU0009 (19.1) *Phaeobacter gallaeciensis*
	WD71	5373 ± 2577.2 *Ν =* 3	288 ± 49.7	351 ± 43.5	0.94 ± 0.026	3.95 ± 0.252	45 (80.2)	OTU0008 (14.2) *Dokdonia donghaensis*
Rotifers	RT	4245 ± 1281.1 *Ν =* 3	282 ± 42	392 ± 80.4	0.93 ± 0.010	3.86 ± 0.150	46 (80.0)	OTU0006 (17.0) *Ruegeria mobilis*
*Artemia* sp. Nauplii	AN	14,363 ± 2993 *Ν =* 3	137 ± 6.6	154 ± 8.9	0.89 ± 0.004	2.98 ± 0.039	13 (80.8)	OTU0004 (23.7) *Alteromonas macleodii*
*Artemia* sp. Metanauplii	AM	9002 ± 1251.6 *Ν =* 3	224 ± 14.0	263 ± 18.5	0.87 ± 0.010	3.22 ± 0.089	21 (80.3)	OTU0005 (31.8) *Phaeobacter italicus*
Commercial diet	CD	2266 ± 1007.7 *Ν =* 6	257 ± 36.0	349 ± 55.1	0.94 ± 0.031	4.11 ± 0.349	99 (80.1)	OTU0025 (17.0) *Photobacterium phosphoreum*

**Table 2:** The *t*-values (italics) and *p*-values (underlined) of Tukey’s test for the detection of statistically significant differences of the operational taxonomic units (OTU) in *Sparus aurata* larvae from day (D) zero to day 71. *: *p <* 0.05, **: *p <* 0.002. *p*-values are corrected for multiple comparisons.

	**D0**	**D5**	**D15**	**D21**	**D71**
**D0**		0.295	0.626	0.034 *	0.065
**D5**	*2.747*		0.007 **	0.000 **	0.000 **
**D15**	*1.983*	*4.731*		0.587	0.735
**D21**	*4.052*	*6.799*	*2.069*		0.999
**D71**	*3.721*	*6.468*	*1.738*	*0.331*	

## Supplementary Materials

The following are available online at https://www.mdpi.com/2073-4425/10/7/483/s1, Table S1: Individual sequence reads of the investigated developmental stages of *Sparus aurata* larvae. Table S2: Comparison of rarefied (R) and non-rarefied (NR) data performance of the bacterial operational taxonomic units (OTUs) in all the investigated developmental stages of *Sparus aurata* larvae. Figure S1: Rarefaction curves of the sequencing reads in all individual samples, Figure S2: Clustering of bacterial communities at the operational taxonomic units level *Sparus aurata* larvae and the surrounding environment microbial populations. UPGMA clustering was performed on weighted and unweighted UNIFRAC measures between samples based on phylogenetic and OTU distribution distance matrices, Figure S3: Venn diagram of the shared operational taxonomic units between all the investigated developmental stages of *Sparus aurata* larvae, Figure S4: Mean relative abundance of proteobacterial classes in all sample categories (Sparus aurata larvae, rearing water and feeds) D: Day, AM: *Artemia salina* metanauplii, AN: *Artemia salina* nauplii, CD: Commercial diet, RT: Rotifers, W: Water sample., Figure S5: Percentage of shared operational taxonomic units between *Sparus aurata* larvae and the surrounding environment microbial populations.

## References

[1] Valdes A.M., Walter J., Segal E., Spector T.D. (2018). Role of the gut microbiota in nutrition and health. BMJ.

[2] Tissier H. (1900). Recherches Sur la Flore Intestinale des Nourrissons (État Normal et Pathologique).

[3] Salminen S., Gibson G.R., McCartney A.L., Isolauri E. (2004). Influence of mode of delivery on gut microbiota composition in seven year old children. Gut.

[4] Biasucci G., Benenati B., Morelli L., Bessi E., Boehm G. (2008). Cesarean delivery may affect the early biodiversity of intestinal bacteria. J. Nutr..

[5] Jiménez E., Marín M.L., Martín R., Odriozola J.M., Olivares M., Xaus J., Fernández L., Rodríguez J.M. (2008). Is meconium from healthy newborns actually sterile?. Res. Microbiol..

[6] Gosalbes M.J., Llop S., Valles Y., Moya A., Ballester F., Francino M.P. (2013). Meconium microbiota types dominated by lactic acid or enteric bacteria are differentially associated with maternal eczema and respiratory problems in infants. Clin. Exp. Allergy J. Br. Soc. Allergy Clin. Immunol..

[7] Perez-Muñoz M.E., Arrieta M.-C., Ramer-Tait A.E., Walter J. (2017). A critical assessment of the “sterile womb” and “in utero colonization” hypotheses: Implications for research on the pioneer infant microbiome. Microbiome.

[8] Sprockett D., Fukami T., Relman D.A. (2018). Role of priority effects in the early-life assembly of the gut microbiota. Nat. Rev. Gastroenterol. Hepatol..

[9] Van Best N., Hornef M.W., Savelkoul P.H., Penders J. (2015). On the origin of species: Factors shaping the establishment of infant′s gut microbiota. Birth Defects Res. Part C Embryo Today Rev..

[10] Zorriehzahra M.J., Delshad S.T., Adel M., Tiwari R., Karthik K., Dhama K., Lazado C.C. (2016). Probiotics as beneficial microbes in aquaculture: An update on their multiple modes of action: A review. Vet. Q..

[11] Hansen G.H., Olafsen J.A. (1999). Bacterial interactions in early life stages of marine cold water fish. Microb. Ecol..

[12] Olafsen J.A. (2001). Interactions between fish larvae and bacteria in marine aquaculture. Aquaculture.

[13] Olafsen J.A., DahI E., Danielssen D.S., Moksness E., Solemdal P. (1984). Ingestion of bacteria by cod (*Gadus morhua* L.) larvae. The Propagation of Cod Gadus morhua L..

[14] Olafsen J.A., Hansen G.H. (1992). Intact antigen uptake in intestinal epithelial cells of marine fish larvae. J. Fish Biol..

[15] Beveridge M.C.M., Sikdar P.K., Frerichs G.N., Millar S. (1991). The ingestion of bacteria in suspension by the common carp *Cyprinus carpio* L.. J. Fish Biol..

[16] Sullam K.E., Essinger S.D., Lozupone C.A., O’Connor M.P., Rosen G.L., Knight R.O.B., Kilham S.S., Russell J.A. (2012). Environmental and ecological factors that shape the gut bacterial communities of fish: A meta-analysis. Mol. Ecol..

[17] Parris D.J., Brooker R.M., Morgan M.A., Dixson D.L., Stewart F.J. (2016). Whole gut microbiome composition of damselfish and cardinalfish before and after reef settlement. PeerJ.

[18] Zhang Z., Li D. (2018). Thermal processing of food reduces gut microbiota diversity of the host and triggers adaptation of the microbiota: Evidence from two vertebrates. Microbiome.

[19] Dehler C.E., Secombes C.J., Martin S.A.M. (2017). Environmental and physiological factors shape the gut microbiota of atlantic salmon parr (*Salmo salar* L.). Aquaculture.

[20] Vadstein O., Attramadal K.J.K., Bakke I., Forberg T., Olsen Y., Verdegem M., Giatsis C., Skjermo J., Aasen I.M., Gatesoupe F.J. (2018). Managing the microbial community of marine fish larvae: A holistic perspective for larviculture. Front. Microbiol..

[21] Llewellyn M.S., Boutin S., Hoseinifar S.H., Derome N. (2014). Teleost microbiomes: The state of the art in their characterization, manipulation and importance in aquaculture and fisheries. Front. Microbiol..

[22] Floris R., Manca S., Fois N. (2013). Microbial ecology of intestinal tract of gilthead sea bream (*Sparus aurata* Linnaeus, 1758) from two coastal lagoons of Sardinia (Italy). Transit. Waters Bull..

[23] Kormas K.A., Meziti A., Mente E., Frentzos A. (2014). Dietary differences are reflected on the gut prokaryotic community structure of wild and commercially reared sea bream (*Sparus aurata*). Microbiol. Open.

[24] Bourouni O.C., Barros-Velazquez J., Calo-Mata P., El Bour M. (2015). Lactic acid bacteria associated with the digestive tract and skin of sea bream (*Sparus aurata*) cultured in Tunisia. Afr. J. Microbiol. Res..

[25] Nikouli E., Meziti A., Antonopoulou E., Mente E., Kormas K. (2018). Gut bacterial communities in geographically distant populations of farmed sea bream (*Sparus aurata*) and sea bass (*Dicentrarchus labrax*). Microorganisms.

[26] Suzer C., Çoban D., Kamaci H.O., Saka Ş., Firat K., Otgucuoğlu Ö., Küçüksari H. (2008). *Lactobacillus* spp. Bacteria as probiotics in gilthead sea bream (*Sparus aurata*, L.) larvae: Effects on growth performance and digestive enzyme activities. Aquaculture.

[27] Dimitroglou A., Merrifield D.L., Spring P., Sweetman J., Moate R., Davies S.J. (2010). Effects of mannan oligosaccharide (mos) supplementation on growth performance, feed utilisation, intestinal histology and gut microbiota of gilthead sea bream (*Sparus aurata*). Aquaculture.

[28] Silva F.C.d.P., Nicoli J.R., Zambonino-Infante J.L., Kaushik S., Gatesoupe F.-J. (2011). Influence of the diet on the microbial diversity of faecal and gastrointestinal contents in gilthead sea bream (*Sparus aurata*) and intestinal contents in goldfish (*Carassius auratus*). Fems Microbiol. Ecol..

[29] Tapia-Paniagua S.T., Reyes-Becerril M., Ascencio-Valle F., Esteban M.Á., Clavijo E., Balebona M.C., Moriñigo M.A. (2011). Modulation of the intestinal microbiota and immune system of farmed *Sparus aurata* by the administration of the yeast *Debaryomyces hansenii* l2 in conjunction with inulin. J. Aquac. Res. Dev..

[30] Cerezuela R., Fumanal M., Tapia-Paniagua S.T., Meseguer J., Moriñigo M.Á., Esteban M.Á. (2013). Changes in intestinal morphology and microbiota caused by dietary administration of inulin and bacillus subtilis in gilthead sea bream (*Sparus aurata* L.) specimens. Fish Shellfish Immunol..

[31] Estruch G., Collado M.C., Peñaranda D.S., Tomás Vidal A., Jover Cerdá M., Pérez Martínez G., Martinez-Llorens S. (2015). Impact of fishmeal replacement in diets for gilthead sea bream *Sparus aurata* on the gastrointestinal microbiota determined by pyrosequencing the 16S rRNA gene. PLoS ONE.

[32] Parma L., Candela M., Soverini M., Turroni S., Consolandi C., Brigidi P., Mandrioli L., Sirri R., Fontanillas R., Gatta P.P. (2016). Next-generation sequencing characterization of the gut bacterial community of gilthead sea bream (*Sparus aurata*, L.) fed low fishmeal based diets with increasing soybean meal levels. Anim. Feed Sci. Technol..

[33] Grisez L., Reyniers J., Verdonck L., Swings J., Ollevier F. (1997). Dominant intestinal microflora of sea bream and sea bass larvae, from two hatcheries, during larval development. Aquaculture.

[34] Savaş S., Kubilay A., Basmaz N. (2005). Effect of bacterial load in feeds on intestinal microflora of seabream (sparus aurata) larvae and juveniles. Isr. J. Aquac. Bamidgeh.

[35] Califano G., Castanho S., Soares F., Ribeiro L., Cox C.J., Mata L., Costa R. (2017). Molecular taxonomic profiling of bacterial communities in a gilthead sea bream (*Sparus aurata*) hatchery. Front. Microbiol..

[36] Moretti A., Pedini Fernandez-Criado M., Cittolin G., Guidastri R. (1999). Manual on Hatchery Production of Seabass and Gilthead Seabream.

[37] Moretti A., Pedini Fernandez-Criado M., Vetillart R. (2005). Manual on Hatchery Production of Seabass and Gilthead Seabream.

[38] Klindworth A., Pruesse E., Schweer T., Peplies J., Quast C., Horn M., Glöckner F.O. (2012). Evaluation of general 16S ribosomal rna gene pcr primers for classical and next-generation sequencing-based diversity studies. Nucleic Acids Res..

[39] Dowd S., Callaway T., Wolcott R., Sun Y., McKeehan T., Hagevoort R., Edrington T. (2008). Evaluation of the bacterial diversity in the feces of cattle using 16S rDNA bacterial tag-encoded flx amplicon pyrosequencing (btefap). BMC Microbiol..

[40] Schloss P.D., Westcott S.L., Ryabin T., Hall J.R., Hartmann M., Hollister E.B., Lesniewski R.A., Oakley B.B., Parks D.H., Robinson C.J. (2009). Introducing mothur: Open-source, platform-independent, community-supported software for describing and comparing microbial communities. Appl. Environ. Microbiol..

[41] Schloss P.D., Gevers D., Westcott S.L. (2011). Reducing the effects of pcr amplification and sequencing artifacts on 16S rRNA-based studies. PLoS ONE.

[42] Pruesse E., Peplies J., Glöckner F.O. (2012). Sina: Accurate high-throughput multiple sequence alignment of ribosomal rna genes. Bioinformatics.

[43] Quast C., Pruesse E., Yilmaz P., Gerken J., Schweer T., Yarza P., Peplies J., Glöckner F.O. (2013). The SILVA ribosomal RNA gene database project: Improved data processing and web-based tools. Nucleic Acids Res..

[44] Hammer Ø., Harper D., Ryan P. (2001). Past: Paleontological statistics software package for education and data analysis. Palaeontol. Electr..

[45] Rstudio Team Integrated Development for R. Rstudio, Inc., Boston, Ma. http://www.rstudio.com/.

[46] McFall-Ngai M., Hadfield M.G., Bosch T.C.G., Carey H.V., Domazet-Lošo T., Douglas A.E., Dubilier N., Eberl G., Fukami T., Gilbert S.F. (2013). Animals in a bacterial world, a new imperative for the life sciences. Proc. Natl. Acad. Sci. USA.

[47] Yatsunenko T., Rey F.E., Manary M.J., Trehan I., Dominguez-Bello M.G., Contreras M., Magris M., Hidalgo G., Baldassano R.N., Anokhin A.P. (2012). Human gut microbiome viewed across age and geography. Nature.

[48] Hansen G.H., Olafsen J.A. (1989). Bacterial colonization of cod (*Gadus morhua* L.) and halibut (*Hippoglossus hippoglossus*) eggs in marine aquaculture. Appl. Environ. Microbiol..

[49] Tytler P., Blaxter J.H.S. (1988). Drinking in yolk-sac stage larvae of the halibut, *Hippoglossus hippoglossus* (L.). J. Fish Biol..

[50] Muroga K., Higashi M., Keitoku H. (1987). The isolation of intestinal microflora of farmed red seabream (*Pagrus major*) and black seabream (*Acanthopagrus schlegeli*) at larval and juvenile stages. Aquaculture.

[51] Tanasomwang V., Muroga K. (1988). Intestinal microflora of larval and juvenile stages in japanese flounder (*Paralichthys olivaceus*). Fish Pathol..

[52] Ingerslev H.C., von Gersdorff Jørgensen L., Lenz Strube M., Larsen N., Dalsgaard I., Boye M., Madsen L. (2014). The development of the gut microbiota in rainbow trout (*Oncorhynchus mykiss*) is affected by first feeding and diet type. Aquaculture.

[53] Bakke I., Skjermo J., Vo T.A., Vadstein O. (2013). Live feed is not a major determinant of the microbiota associated with cod larvae (*Gadus morhua*). Environ. Microbiol. Rep..

[54] Bates J.M., Mittge E., Kuhlman J., Baden K.N., Cheesman S.E., Guillemin K. (2006). Distinct signals from the microbiota promote different aspects of zebrafish gut differentiation. Dev. Biol..

[55] Muroga K., Yasunobu H. (1987). Uptake of bacteria by rotifer. Nippon Suisan Gakkaishi.

[56] Munro P.D., Birkbeck T.H., Barbour A., Reinertsen H., Dahle L.A., Jorgensen L., Tvinnereim K. (1993). Influence of rate of bacterial colonization of the gut of turbot larvae on larval survival. Fish Farming Technology.

[57] Skjermo J., Vadstein O. (1993). Characterization of the bacterial flora of mass cultivated *Brachionus plicatilis*. Hydrobiologia.

[58] Ishino R., Iehata S., Nakano M., Tanaka R., Yoshimatsu T., Maeda H. (2012). Bacterial diversity associated with the rotifer *Brachionus plicatilis* sp. Complex determined by culture-dependent and -independent methods. Biocontrol Sci..

[59] McHenery J.G., Birkbeck T.H. (1985). Uptake and processing of cultured microorganisms by bivalves. J. Exp. Mar. Biol. Ecol..

[60] Stephens W.Z., Burns A.R., Stagaman K., Wong S., Rawls J.F., Guillemin K., Bohannan B.J.M. (2016). The composition of the zebrafish intestinal microbial community varies across development. ISME J..

[61] Rombout J.H.W.M., Abelli L., Picchietti S., Scapigliati G., Kiron V. (2011). Teleost intestinal immunology. Fish Shellfish Immunol..

[62] Tarnecki A.M., Burgos F.A., Ray C.L., Arias C.R. (2017). Fish intestinal microbiome: Diversity and symbiosis unraveled by metagenomics. J. Appl. Microbiol..

[63] Ghanbari M., Kneifel W., Domig K.J. (2015). A new view of the fish gut microbiome: Advances from next-generation sequencing. Aquaculture.

[64] Parte A.C. (2018). Lpsn—list of prokaryotic names with standing in nomenclature (bacterio.Net), 20 years on. Int. J. Syst. Evol. Microbiol..

[65] Hansen G.H., Bergh O., Michaelsen J., Knappskog D. (1992). *Flexibacter ovolyticus* sp. nov., a pathogen of eggs and larvae of atlantic halibut, *Hippoglossus hippoglossus* L.. Int. J. Syst. Bacteriol..

[66] Piñeiro-Vidal M., Riaza A., Santos Y. (2008). Tenacibaculum discolor sp. nov. and Tenacibaculum gallaicum sp. nov., isolated from sole (Solea senegalensis) and turbot (Psetta maxima) culture systems. Int. J. Syst. Evol. Microbiol..

[67] Avendaño-Herrera R., Irgang R., Sandoval C., Moreno-Lira P., Houel A., Duchaud E., Poblete-Morales M., Nicolas P., Ilardi P. (2016). Isolation, characterization and virulence potential of *Tenacibaculum dicentrarchi* in salmonid cultures in chile. Transbound. Emerg. Dis..

[68] Olsen A.B., Gulla S., Steinum T., Colquhoun D.J., Nilsen H.K., Duchaud E. (2017). Multilocus sequence analysis reveals extensive genetic variety within *Tenacibaculum* spp. associated with ulcers in sea-farmed fish in norway. Vet. Microbiol..

[69] Yoon J., Kasai H., Yokota A. (2010). Phylogenetic interrelationships of the genus *Rubritalea* inferred from 16S rRNA and *gyrb* gene sequences. Microbiol. Cult. Collect..

[70] Dishaw L.J., Flores-Torres J., Lax S., Gemayel K., Leigh B., Melillo D., Mueller M.G., Natale L., Zucchetti I., De Santis R. (2014). The gut of geographically disparate *Ciona intestinalis* harbors a core microbiota. PLoS ONE.

[71] Mandakovic D., Glasner B., Maldonado J., Aravena P., González M., Cambiazo V., Pulgar R. (2016). Genomic-based restriction enzyme selection for specific detection of *Piscirickettsia salmonis* by 16S rDNA PCR-RFLP. Front. Microbiol..

[72] Muramatsu Y., Uchino Y., Kasai H., Suzuki K., Nakagawa Y. (2007). *Ruegeria mobilis* sp. nov., a member of the Alphaproteobacteria isolated in Japan and Palau. Int. J. Syst. Evol. Microbiol..

[73] D’Alvise P.W., Magdenoska O., Melchiorsen J., Nielsen K.F., Gram L. (2014). Biofilm formation and antibiotic production in *Ruegeria mobilis* are influenced by intracellular concentrations of cyclic dimeric guanosinmonophosphate. Environ. Microbiol..

[74] D’Alvise P.W., Lillebø S., Prol-Garcia M.J., Wergeland H.I., Nielsen K.F., Bergh Ø., Gram L. (2012). *Phaeobacter gallaeciensis* reduces *Vibrio anguillarum* in cultures of microalgae and rotifers, and prevents vibriosis in cod larvae. PLoS ONE.

[75] Ruby E.G., Morin J.G. (1979). Luminous enteric bacteria of marine fishes: A study of their distribution, densities, and dispersion. Appl. Environ. Microbiol..

[76] Hovda M.B., Lunestad B.T., Fontanillas R., Rosnes J.T. (2007). Molecular characterisation of the intestinal microbiota of farmed Atlantic salmon (*Salmo salar* L.). Aquaculture.

