# Supplementary material for: Host-Associated Bacterial Succession during the Early Embryonic Stages and First Feeding in Farmed Gilthead Sea Bream (Sparus aurata)
Source: Genes (Basel). 2019 Jun 26;10(7):483. doi: 10.3390/genes10070483 (PMC6678923; doi:10.3390/genes10070483)
Supplement: Supplementary file 1 [file genes-10-00483-s001.pdf]

Host-Associated Bacterial Succession during the Early Embryonic Stages and First Feeding in Farmed Gilthead Sea Bream (*Sparus aurata*)

Eleni Nikouli [1], Alexandra Meziti [1], Efthimia Antonopoulou [2], Eleni Mente [1] and Konstantinos Ar. Kormas [1,*]

[1] Department of Ichthyology and Aquatic Environment, School of Agricultural Sciences, University of Thessaly, Volos 384 46, Greece; EN: elnikoul@uth.gr; AM: ameziti@gmail.com; EM: emente@uth.gr

[2] Laboratory of Animal Physiology, Department of Zoology, School of Biology, Aristotle University of Thessaloniki, 541 24, Greece; eantono@bio.auth.gr

* Correspondence: kkormas@uth.gr, kkormas@gmail.com; Tel.: +30-242-109-3082

Supplementary material

Table S1. Individual sequence reads of the investigated developmental stages of *Sparus aurata* larvae. OTU: operational taxonomic unit; D: day; a, b, c: replicate sample.

	**D0a**	**D0b**	**D0c**		**D5a**	**D5b**		**D15a**	**D15b**	**D15c**		**D21a**	**D21b**	**D21c**		**D71a**	**D71b**
OTU_26	586	1082	147	*OTU_7*	1959	2023	*OTU_6*	554	1398	457	*OTU_6*	55	415	42	*OTU_25*	14	151
OTU_11	943	241	84	*OTU_12*	1903	774	*OTU_42*	64	340	42	*OTU_9*	78	137	72	*OTU_101*	27	111
OTU_24	53	182	778	*OTU_11*	99	2025	*OTU_89*	173	212	61	*OTU_5*	140	85	48	*OTU_94*	14	99
OTU_32	6	30	892	*OTU_532*	103	503	*OTU_156*	54	261	29	*OTU_1913*	14	36	104	*OTU_355*	75	27
OTU_47	0	11	617	*OTU_942*	301	272	*OTU_97*	299	41	0	*OTU_89*	13	109	9	*OTU_20*	73	25
OTU_52	15	26	314	*OTU_58*	101	452	*OTU_56*	58	59	159	*OTU_66*	38	23	40	*OTU_1616*	47	9
OTU_113	8	18	301	*OTU_354*	255	261	*OTU_737*	113	117	20	*OTU_194*	15	1	78	*OTU_168*	0	53
OTU_152	0	8	258	*OTU_1089*	116	110	*OTU_66*	81	95	56	*OTU_57*	6	54	25	*OTU_263*	0	53
OTU_20	30	208	20	*OTU_121*	163	3	*OTU_40*	0	216	0	*OTU_122*	35	15	22	*OTU_87*	8	44
OTU_12	10	180	27	*OTU_23*	46	100	*OTU_1913*	142	51	21	*OTU_37*	13	39	16	*OTU_436*	40	10
OTU_23	13	189	4	*OTU_1794*	75	69	*OTU_493*	78	110	15	*OTU_60*	5	21	24	*OTU_179*	0	44
OTU_82	19	178	1	*OTU_944*	109	23	*OTU_43*	59	79	7	*OTU_46*	8	27	14	*OTU_5*	6	37
OTU_10	47	115	23	*OTU_1561*	67	60	*OTU_172*	11	86	18	*OTU_108*	2	10	30	*OTU_212*	17	24
OTU_214	4	159	13	*OTU_191*	64	57	*OTU_1821*	43	65	6	*OTU_42*	3	23	9	*OTU_945*	25	15
OTU_104	0	0	154	*OTU_193*	58	51	*OTU_60*	13	78	12	*OTU_1215*	7	26	1	*OTU_240*	0	39
OTU_110	0	0	147	*OTU_331*	63	41	*OTU_63*	41	49	2	*OTU_67*	1	9	23	*OTU_29*	1	37
OTU_109	0	2	143	*OTU_524*	8	81	*OTU_326*	43	5	35	*OTU_26*	4	19	6	*OTU_218*	0	38
OTU_356	44	84	10	*OTU_1552*	35	48	*OTU_57*	44	4	34	*OTU_172*	8	10	11	*OTU_403*	0	37
OTU_123	12	20	95	*OTU_82*	40	38	*OTU_382*	35	42	1	*OTU_54*	9	8	9	*OTU_53*	23	13
OTU_90	8	74	37	*OTU_1341*	13	61	*OTU_277*	10	54	10	*OTU_10*	7	8	10	*OTU_35*	0	34
OTU_120	0	0	119	*OTU_665*	10	63	*OTU_1215*	2	46	25	*OTU_1632*	4	8	13	*OTU_136*	11	23
OTU_300	3	2	114	*OTU_847*	27	46	*OTU_26*	28	40	3	*OTU_592*	0	19	4	*OTU_298*	0	34
OTU_1437	32	76	10	*OTU_413*	29	42	*OTU_22*	2	60	0	*OTU_1820*	0	22	0	*OTU_1738*	30	3
OTU_140	4	50	54	*OTU_773*	27	44	*OTU_1008*	29	9	23	*OTU_213*	1	20	0	*OTU_334*	32	0
OTU_223	29	76	3	*OTU_1867*	36	32	*OTU_1699*	16	19	17	*OTU_277*	0	4	17	*OTU_65*	0	30
OTU_1848	0	2	102	*OTU_443*	37	29	*OTU_407*	34	15	2	*OTU_22*	3	4	13	*OTU_4*	11	17

OTU_18	78	6	7	*OTU_688*	2	39	*OTU_4*	8	34	7	*OTU_40*	7	6	7	*OTU_129*	8	20
OTU_147	0	0	90	*OTU_597*	39	0	*OTU_652*	19	26	4	*OTU_407*	5	9	6	*OTU_341*	0	26
OTU_125	33	6	49	*OTU_61*	16	17	*OTU_872*	27	16	4	*OTU_69*	0	13	6	*OTU_148*	3	22
OTU_592	3	3	80	*OTU_1474*	14	19	*OTU_5*	12	9	24	*OTU_41*	1	8	8	*OTU_353*	0	25
OTU_149	0	5	75	*OTU_1563*	5	28	*OTU_1478*	16	23	2	*OTU_106*	9	3	5	*OTU_128*	0	23
OTU_146	5	52	13	*OTU_10*	16	13	*OTU_54*	12	16	9	*OTU_1319*	2	6	9	*OTU_392*	0	23
OTU_162	27	39	2	*OTU_142*	25	1	*OTU_194*	1	35	1	*OTU_23*	0	16	0	*OTU_6*	22	0
OTU_4	3	62	0	*OTU_1033*	2	20	*OTU_131*	18	17	1	*OTU_156*	6	9	1	*OTU_266*	0	22
OTU_208	0	0	63	*OTU_59*	20	0	*OTU_129*	4	25	6	*OTU_1809*	0	16	0	*OTU_1506*	2	20
OTU_246	0	0	63	*OTU_1808*	5	14	*OTU_554*	6	28	0	*OTU_56*	6	1	8	*OTU_1632*	22	0
OTU_50	55	5	2	*OTU_994*	14	4	*OTU_612*	11	22	1	*OTU_1145*	4	0	11	*OTU_476*	15	6
OTU_281	4	53	0	*OTU_207*	9	8	*OTU_1809*	6	26	2	*OTU_197*	0	14	0	*OTU_1870*	21	0
OTU_129	1	52	0	*OTU_1658*	7	10	*OTU_1319*	28	0	4	*OTU_236*	0	1	13	*OTU_21*	1	19
OTU_190	23	25	4	*OTU_1421*	5	10	*OTU_281*	1	26	4	*OTU_1870*	11	0	3	*OTU_281*	5	15
OTU_227	0	4	44	*OTU_229*	14	0	*OTU_1051*	23	6	1	*OTU_43*	2	8	3	*OTU_460*	0	17
OTU_374	0	0	47	*OTU_2*	11	2	*OTU_1322*	13	9	7	*OTU_1887*	1	10	2	*OTU_10*	0	16
OTU_432	11	12	23	*OTU_312*	7	6	*OTU_1145*	19	7	0	*OTU_586*	0	1	11	*OTU_239*	15	1
OTU_1730	0	1	44	*OTU_1576*	0	12	*OTU_82*	11	4	10	*OTU_1774*	0	6	6	*OTU_470*	15	1
OTU_524	26	11	7	*OTU_107*	8	3	*OTU_184*	15	8	2	*OTU_554*	0	1	10	*OTU_481*	0	16
OTU_278	0	0	42	*OTU_346*	11	0	*OTU_248*	4	7	14	*OTU_836*	2	7	2	*OTU_1725*	10	6
OTU_473	0	9	32	*OTU_922*	6	5	*OTU_342*	18	5	2	*OTU_24*	8	2	0	*OTU_41*	0	15
OTU_256	0	0	40	*OTU_1179*	0	11	*OTU_1462*	3	17	5	*OTU_180*	1	1	8	*OTU_202*	15	0
OTU_6	0	24	15	*OTU_451*	9	1	*OTU_1747*	21	4	0	*OTU_604*	0	6	4	*OTU_431*	0	15
OTU_1068	0	0	39	*OTU_500*	10	0	*OTU_364*	19	0	5	*OTU_646*	0	2	8	*OTU_998*	6	9
OTU_1808	3	36	0	*OTU_1056*	9	0	*OTU_122*	14	4	5	*OTU_1534*	5	2	3	*OTU_402*	0	14
OTU_232	17	17	3	*OTU_215*	4	4	*OTU_9*	0	10	12	*OTU_1852*	1	3	6	*OTU_667*	0	14
OTU_51	0	34	0	*OTU_239*	8	0	*OTU_908*	1	21	0	*OTU_131*	6	1	2	*OTU_682*	14	0
OTU_287	0	0	34	*OTU_264*	0	8	*OTU_1776*	20	1	0	*OTU_192*	1	0	8	*OTU_426*	13	0
OTU_1145	5	28	1	*OTU_658*	2	6	*OTU_1785*	2	17	2	*OTU_250*	5	0	4	*OTU_455*	7	6
OTU_69	0	0	33	*OTU_832*	4	4	*OTU_1911*	3	17	1	*OTU_261*	4	2	3	*OTU_619*	13	0

OTU_420	2	31	0	*OTU_28*	3	4	*OTU_106*	15	3	1	*OTU_365*	0	4	5	*OTU_84*	0	12
OTU_903	0	3	29	*OTU_1837*	4	3	*OTU_715*	4	14	1	*OTU_612*	0	6	3	*OTU_114*	1	11
OTU_61	0	30	0	*OTU_22*	5	1	*OTU_753*	0	15	4	*OTU_1014*	1	8	0	*OTU_177*	0	12
OTU_307	0	30	0	*OTU_44*	6	0	*OTU_1820*	0	18	1	*OTU_4*	0	2	6	*OTU_181*	12	0
OTU_323	0	0	30	*OTU_1409*	5	1	*OTU_548*	11	3	4	*OTU_20*	0	7	1	*OTU_258*	12	0
OTU_1275	0	0	30	*OTU_1437*	3	3	*OTU_1380*	18	0	0	*OTU_125*	0	8	0	*OTU_360*	8	4
OTU_1507	0	0	29	*OTU_1870*	6	0	*OTU_772*	7	9	1	*OTU_427*	1	6	1	*OTU_390*	0	12
OTU_242	0	27	0	*OTU_225*	5	0	*OTU_64*	16	0	0	*OTU_434*	8	0	0	*OTU_440*	12	0
OTU_312	8	18	1	*OTU_448*	0	5	*OTU_759*	10	5	1	*OTU_485*	0	8	0	*OTU_492*	2	10
OTU_351	0	0	27	*OTU_726*	0	5	*OTU_1350*	0	16	0	*OTU_493*	0	5	3	*OTU_542*	0	12
OTU_688	17	8	1	*OTU_1119*	3	2	*OTU_1522*	12	1	3	*OTU_1699*	0	5	3	*OTU_611*	11	0
OTU_361	0	10	15	*OTU_1834*	4	1	*OTU_108*	13	0	2	*OTU_1895*	3	4	1	*OTU_937*	1	10
OTU_944	1	22	2	*OTU_1885*	0	5	*OTU_297*	3	12	0	*OTU_15*	1	4	2	*OTU_122*	5	5
OTU_1339	2	1	22	*OTU_38*	3	1	*OTU_1632*	14	0	1	*OTU_63*	0	0	7	*OTU_180*	10	0
OTU_1642	0	6	19	*OTU_218*	1	3	*OTU_94*	0	14	0	*OTU_243*	0	5	2	*OTU_502*	10	0
OTU_1870	2	23	0	*OTU_574*	4	0	*OTU_626*	8	5	1	*OTU_281*	3	0	4	*OTU_673*	0	10
OTU_102	0	7	17	*OTU_648*	3	1	*OTU_1287*	7	5	1	*OTU_324*	2	3	2	*OTU_1799*	8	2
OTU_375	0	0	24	*OTU_1145*	2	2	*OTU_1506*	6	3	4	*OTU_424*	0	3	4	*OTU_106*	8	1
OTU_391	0	23	0	*OTU_1516*	4	0	*OTU_24*	12	0	0	*OTU_759*	2	5	0	*OTU_330*	9	0
OTU_415	0	23	0	*OTU_695*	0	3	*OTU_1165*	6	4	2	*OTU_1510*	1	2	4	*OTU_365*	9	0
OTU_44	0	14	7	*OTU_776*	2	1	*OTU_1255*	7	5	0	*OTU_1785*	0	6	1	*OTU_702*	0	9
OTU_94	6	10	5	*OTU_1012*	0	3	*OTU_1738*	3	7	2	*OTU_1821*	0	7	0	*OTU_854*	0	9
OTU_22	3	14	3	*OTU_1303*	1	2	*OTU_359*	0	3	8	*OTU_173*	0	0	6	*OTU_856*	0	9
OTU_417	0	0	20	*OTU_1448*	1	2	*OTU_20*	8	2	0	*OTU_737*	2	3	1	*OTU_882*	9	0
OTU_419	0	10	10	*OTU_1614*	2	1	*OTU_631*	10	0	0	*OTU_753*	0	6	0	*OTU_900*	0	9
OTU_469	3	1	16	*OTU_148*	2	0	*OTU_653*	0	10	0	*OTU_829*	1	3	2	*OTU_972*	8	1
OTU_1453	0	12	8	*OTU_294*	0	2	*OTU_706*	10	0	0	*OTU_1051*	1	4	1	*OTU_51*	0	8
OTU_1629	0	1	19	*OTU_444*	1	1	*OTU_1510*	9	0	1	*OTU_1104*	1	1	4	*OTU_139*	6	2
OTU_101	7	10	2	*OTU_1099*	1	1	*OTU_1712*	1	8	1	*OTU_1747*	1	3	2	*OTU_229*	7	1
OTU_446	0	12	7	*OTU_1183*	2	0	*OTU_1774*	0	10	0	*OTU_51*	0	3	2	*OTU_384*	0	8

OTU_550	1	1	17	*OTU_1202*	1	1	*OTU_304*	6	0	3	*OTU_52*	2	2	1	*OTU_1485*	5	3
OTU_15	0	18	0	*OTU_1480*	1	1	*OTU_10*	2	4	2	*OTU_82*	2	3	0	*OTU_1916*	8	0
OTU_442	0	5	13	*OTU_1581*	2	0	*OTU_219*	8	0	0	*OTU_118*	1	0	4	*OTU_67*	7	0
OTU_512	0	18	0	*OTU_1723*	2	0	*OTU_344*	6	2	0	*OTU_184*	1	2	2	*OTU_324*	0	7
OTU_966	0	7	11	*OTU_1732*	2	0	*OTU_454*	0	7	1	*OTU_325*	0	4	1	*OTU_834*	7	0
OTU_461	0	0	17	*OTU_1805*	0	2	*OTU_1155*	2	6	0	*OTU_336*	0	3	2	*OTU_1313*	4	3
OTU_471	0	0	17	*OTU_1907*	1	1	*OTU_1349*	1	5	2	*OTU_788*	0	3	2	*OTU_1371*	0	7
OTU_1217	1	3	13	*OTU_9*	1	0	*OTU_1495*	8	0	0	*OTU_800*	2	2	1	*OTU_275*	0	6
OTU_1818	0	0	17	*OTU_71*	1	0	*OTU_1616*	5	3	0	*OTU_939*	1	2	2	*OTU_285*	0	6
OTU_188	0	16	0	*OTU_90*	0	1	*OTU_267*	0	0	7	*OTU_1484*	2	1	2	*OTU_312*	0	6
OTU_202	2	14	0	*OTU_144*	1	0	*OTU_838*	7	0	0	*OTU_129*	1	0	3	*OTU_520*	2	4
OTU_239	16	0	0	*OTU_158*	0	1	*OTU_1480*	2	4	1	*OTU_202*	1	0	3	*OTU_603*	2	4
OTU_421	0	7	9	*OTU_199*	1	0	*OTU_312*	4	0	2	*OTU_706*	0	0	4	*OTU_881*	6	0
OTU_491	0	0	16	*OTU_206*	1	0	*OTU_340*	1	5	0	*OTU_772*	1	3	0	*OTU_1645*	0	6
OTU_1738	1	15	0	*OTU_233*	0	1	*OTU_356*	4	2	0	*OTU_842*	0	4	0	*OTU_1646*	3	3
OTU_108	7	8	0	*OTU_394*	0	1	*OTU_427*	5	1	0	*OTU_970*	1	1	2	*OTU_1692*	0	6
OTU_501	0	0	15	*OTU_452*	1	0	*OTU_1017*	6	0	0	*OTU_1183*	0	0	4	*OTU_1882*	1	5
OTU_514	0	15	0	*OTU_575*	1	0	*OTU_1260*	5	1	0	*OTU_1196*	0	0	4	*OTU_15*	5	0
OTU_756	0	15	0	*OTU_630*	0	1	*OTU_1488*	0	6	0	*OTU_1478*	1	2	1	*OTU_16*	0	5
OTU_1861	0	14	1	*OTU_807*	1	0	*OTU_1696*	6	0	0	*OTU_1522*	3	0	1	*OTU_54*	3	2
OTU_346	11	3	0	*OTU_842*	0	1	*OTU_1887*	3	0	3	*OTU_1896*	1	2	1	*OTU_89*	5	0
OTU_554	7	5	2	*OTU_860*	0	1	*OTU_139*	1	3	1	*OTU_12*	0	3	0	*OTU_225*	5	0
OTU_559	4	9	1	*OTU_1001*	1	0	*OTU_148*	0	5	0	*OTU_64*	0	3	0	*OTU_646*	5	0
OTU_1632	5	7	2	*OTU_1062*	1	0	*OTU_223*	2	2	1	*OTU_73*	0	0	3	*OTU_987*	1	4
OTU_43	1	12	0	*OTU_1120*	0	1	*OTU_266*	0	5	0	*OTU_94*	3	0	0	*OTU_1042*	0	5
OTU_260	0	13	0	*OTU_1267*	0	1	*OTU_484*	1	3	1	*OTU_382*	1	1	1	*OTU_1145*	0	5
OTU_266	13	0	0	*OTU_1280*	0	1	*OTU_531*	1	0	4	*OTU_467*	0	0	3	*OTU_1349*	4	1
OTU_505	0	0	13	*OTU_1373*	0	1	*OTU_557*	0	4	1	*OTU_531*	0	0	3	*OTU_1624*	4	1
OTU_1033	10	3	0	*OTU_1482*	1	0	*OTU_788*	0	5	0	*OTU_596*	0	2	1	*OTU_33*	0	4
OTU_1349	0	13	0	*OTU_1545*	0	1	*OTU_836*	3	2	0	*OTU_621*	3	0	0	*OTU_37*	4	0

OTU_1514	0	13	0	*OTU_1585*	0	1	*OTU_868*	4	1	0	*OTU_652*	0	1	2	*OTU_297*	4	0
OTU_1907	1	11	1	*OTU_1632*	0	1	*OTU_1074*	0	5	0	*OTU_851*	0	3	0	*OTU_407*	2	2
OTU_237	0	11	1	*OTU_1742*	0	1	*OTU_1412*	0	5	0	*OTU_908*	0	1	2	*OTU_494*	0	4
OTU_304	11	0	1				*OTU_1892*	1	4	0	*OTU_1225*	0	0	3	*OTU_1071*	4	0
OTU_605	0	12	0				*OTU_153*	1	1	2	*OTU_1271*	0	1	2	*OTU_1163*	0	4
OTU_614	0	4	8				*OTU_173*	0	0	4	*OTU_1291*	0	3	0	*OTU_1267*	0	4
OTU_615	0	0	12				*OTU_186*	0	4	0	*OTU_1361*	1	2	0	*OTU_1270*	4	0
OTU_693	0	0	12				*OTU_225*	0	4	0	*OTU_1411*	3	0	0	*OTU_1325*	4	0
OTU_705	0	0	12				*OTU_239*	0	1	3	*OTU_1462*	1	2	0	*OTU_1724*	4	0
OTU_1614	4	8	0				*OTU_320*	0	4	0	*OTU_1480*	1	0	2	*OTU_205*	3	0
OTU_64	0	11	0				*OTU_560*	0	4	0	*OTU_1670*	0	1	2	*OTU_336*	3	0
OTU_330	0	11	0				*OTU_595*	2	2	0	*OTU_1892*	0	3	0	*OTU_453*	0	3
OTU_390	0	0	11				*OTU_823*	1	2	1	*OTU_186*	0	1	1	*OTU_838*	3	0
OTU_448	3	2	6				*OTU_881*	2	2	0	*OTU_233*	0	1	1	*OTU_855*	3	0
OTU_618	0	0	11				*OTU_1440*	2	2	0	*OTU_263*	1	0	1	*OTU_902*	3	0
OTU_624	0	0	11				*OTU_1771*	0	4	0	*OTU_312*	2	0	0	*OTU_1143*	0	3
OTU_685	0	0	11				*OTU_1852*	3	0	1	*OTU_330*	1	0	1	*OTU_1177*	0	3
OTU_718	0	9	2				*OTU_1898*	4	0	0	*OTU_416*	1	0	1	*OTU_1309*	0	3
OTU_766	2	8	1				*OTU_16*	2	1	0	*OTU_476*	0	0	2	*OTU_1315*	3	0
OTU_1485	0	11	0				*OTU_17*	0	0	3	*OTU_523*	0	0	2	*OTU_1382*	0	3
OTU_98	6	0	4				*OTU_45*	0	3	0	*OTU_557*	0	2	0	*OTU_1480*	2	1
OTU_148	1	1	8				*OTU_71*	0	3	0	*OTU_560*	0	0	2	*OTU_1484*	3	0
OTU_360	10	0	0				*OTU_233*	2	1	0	*OTU_606*	1	1	0	*OTU_1522*	1	2
OTU_369	2	8	0				*OTU_254*	3	0	0	*OTU_626*	0	2	0	*OTU_1756*	0	3
OTU_467	0	7	3				*OTU_272*	1	2	0	*OTU_631*	1	1	0	*OTU_1798*	0	3
OTU_661	0	0	10				*OTU_329*	0	3	0	*OTU_790*	0	2	0	*OTU_1830*	0	3
OTU_669	10	0	0				*OTU_503*	3	0	0	*OTU_823*	0	2	0	*OTU_77*	2	0
OTU_687	0	10	0				*OTU_592*	2	0	1	*OTU_867*	0	2	0	*OTU_130*	0	2
OTU_1109	0	2	8				*OTU_650*	0	3	0	*OTU_872*	1	1	0	*OTU_184*	2	0
OTU_1228	0	10	0				*OTU_860*	0	3	0	*OTU_880*	0	2	0	*OTU_268*	2	0

OTU_41	0	9	0				OTU_867	0	1	2	OTU_1150	0	1	1	OTU_412	0	2
OTU_229	5	1	3				OTU_1029	3	0	0	OTU_1174	1	1	0	OTU_424	2	0
OTU_483	0	0	9				OTU_1104	2	1	0	OTU_1220	1	1	0	OTU_462	2	0
OTU_585	0	9	0				OTU_1315	2	0	1	OTU_1275	0	1	1	OTU_668	2	0
OTU_589	0	0	9				OTU_1437	2	0	1	OTU_1298	0	0	2	OTU_766	0	2
OTU_720	0	0	9				OTU_1449	3	0	0	OTU_1313	2	0	0	OTU_790	0	2
OTU_722	0	1	8				OTU_1465	1	1	1	OTU_1315	0	0	2	OTU_836	1	1
OTU_806	0	0	9				OTU_1485	0	2	1	OTU_1322	2	0	0	OTU_975	0	2
OTU_902	0	9	0				OTU_1534	0	0	3	OTU_1350	0	0	2	OTU_1137	2	0
OTU_1089	0	8	1				OTU_1565	0	3	0	OTU_1386	0	0	2	OTU_1228	1	1
OTU_1576	8	1	0				OTU_1601	3	0	0	OTU_1430	0	0	2	OTU_1289	0	2
OTU_1804	0	1	8				OTU_1870	1	1	1	OTU_1437	1	0	1	OTU_1376	2	0
OTU_5	0	8	0				OTU_23	2	0	0	OTU_1440	0	0	2	OTU_1392	2	0
OTU_42	2	6	0				OTU_37	2	0	0	OTU_1582	0	1	1	OTU_1394	1	1
OTU_180	0	8	0				OTU_101	0	0	2	OTU_1624	0	0	2	OTU_1437	1	1
OTU_294	8	0	0				OTU_141	0	0	2	OTU_1720	0	2	0	OTU_1453	0	2
OTU_474	0	8	0				OTU_146	2	0	0	OTU_1727	0	0	2	OTU_1565	2	0
OTU_519	0	8	0				OTU_258	0	2	0	OTU_1801	1	1	0	OTU_1762	1	1
OTU_698	0	0	8				OTU_289	1	0	1	OTU_1808	0	1	1	OTU_1852	2	0
OTU_775	0	8	0				OTU_318	0	2	0	OTU_8	1	0	0	OTU_1898	2	0
OTU_789	5	2	1				OTU_321	1	1	0	OTU_21	1	0	0	OTU_1907	1	1
OTU_842	0	1	7				OTU_325	1	1	0	OTU_58	0	1	0	OTU_69	0	1
OTU_970	0	7	1				OTU_336	2	0	0	OTU_71	0	1	0	OTU_82	0	1
OTU_1015	0	3	5				OTU_416	0	2	0	OTU_92	0	0	1	OTU_146	0	1
OTU_1114	3	2	3				OTU_521	2	0	0	OTU_93	0	1	0	OTU_272	0	1
OTU_1122	0	0	8				OTU_576	2	0	0	OTU_97	0	1	0	OTU_321	1	0
OTU_1127	0	7	1				OTU_587	0	0	2	OTU_101	0	0	1	OTU_329	1	0
OTU_1179	5	3	0				OTU_600	2	0	0	OTU_102	1	0	0	OTU_378	0	1
OTU_1267	3	5	0				OTU_709	0	2	0	OTU_113	1	0	0	OTU_454	0	1
OTU_1480	0	8	0				OTU_744	0	1	1	OTU_134	0	0	1	OTU_478	1	0

OTU_121	0	7	0				*OTU_770*	0	2	0	*OTU_148*	0	1	0	*OTU_488*	0	1
OTU_609	0	0	7				*OTU_805*	2	0	0	*OTU_153*	0	0	1	*OTU_517*	0	1
OTU_611	0	7	0				*OTU_813*	0	2	0	*OTU_165*	0	0	1	*OTU_530*	1	0
OTU_796	0	7	0				*OTU_829*	0	0	2	*OTU_207*	0	0	1	*OTU_595*	0	1
OTU_816	0	7	0				*OTU_953*	2	0	0	*OTU_209*	1	0	0	*OTU_596*	0	1
OTU_826	0	7	0				*OTU_985*	1	1	0	*OTU_212*	1	0	0	*OTU_626*	1	0
OTU_907	0	0	7				*OTU_1080*	0	2	0	*OTU_247*	0	0	1	*OTU_641*	0	1
OTU_1077	0	1	6				*OTU_1193*	2	0	0	*OTU_266*	1	0	0	*OTU_706*	1	0
OTU_1617	0	0	7				*OTU_1285*	2	0	0	*OTU_300*	0	0	1	*OTU_751*	0	1
OTU_33	0	6	0				*OTU_1291*	2	0	0	*OTU_326*	0	1	0	*OTU_757*	0	1
OTU_40	0	6	0				*OTU_1466*	1	1	0	*OTU_356*	0	0	1	*OTU_764*	0	1
OTU_114	1	5	0				*OTU_1500*	2	0	0	*OTU_369*	0	0	1	*OTU_772*	1	0
OTU_130	0	0	6				*OTU_1513*	1	1	0	*OTU_436*	1	0	0	*OTU_787*	1	0
OTU_225	0	6	0				*OTU_1524*	0	2	0	*OTU_478*	0	1	0	*OTU_793*	1	0
OTU_263	3	1	2				*OTU_1536*	0	1	1	*OTU_500*	0	0	1	*OTU_914*	1	0
OTU_384	1	4	1				*OTU_1548*	1	1	0	*OTU_503*	0	0	1	*OTU_934*	1	0
OTU_402	0	6	0				*OTU_1645*	2	0	0	*OTU_510*	0	0	1	*OTU_962*	0	1
OTU_438	0	6	0				*OTU_1735*	1	1	0	*OTU_535*	0	0	1	*OTU_1044*	1	0
OTU_476	0	0	6				*OTU_1757*	1	0	1	*OTU_542*	0	0	1	*OTU_1062*	0	1
OTU_557	0	6	0				*OTU_1801*	0	1	1	*OTU_548*	0	1	0	*OTU_1096*	1	0
OTU_699	0	3	3				*OTU_1808*	2	0	0	*OTU_550*	1	0	0	*OTU_1104*	1	0
OTU_938	0	0	6				*OTU_1837*	2	0	0	*OTU_595*	0	1	0	*OTU_1131*	1	0
OTU_964	0	6	0				*OTU_1896*	1	1	0	*OTU_635*	0	0	1	*OTU_1183*	0	1
OTU_982	2	4	0				*OTU_2*	0	0	1	*OTU_650*	0	0	1	*OTU_1226*	1	0
OTU_1016	0	0	6				*OTU_7*	0	1	0	*OTU_718*	0	1	0	*OTU_1255*	1	0
OTU_1028	6	0	0				*OTU_8*	1	0	0	*OTU_722*	0	1	0	*OTU_1275*	1	0
OTU_1043	0	0	6				*OTU_11*	0	1	0	*OTU_744*	1	0	0	*OTU_1337*	0	1
OTU_1064	0	0	6				*OTU_28*	0	0	1	*OTU_763*	1	0	0	*OTU_1342*	1	0
OTU_1175	0	0	6				*OTU_35*	0	0	1	*OTU_785*	0	1	0	*OTU_1380*	1	0
OTU_1183	1	5	0				*OTU_44*	1	0	0	*OTU_838*	0	0	1	*OTU_1428*	0	1

OTU_1202	0	3	3				*OTU_46*	0	1	0	*OTU_845*	0	1	0	*OTU_1433*	1	0
OTU_1311	0	4	2				*OTU_51*	0	0	1	*OTU_905*	0	0	1	*OTU_1435*	1	0
OTU_1505	1	0	5				*OTU_81*	0	1	0	*OTU_923*	0	1	0	*OTU_1487*	1	0
OTU_1589	0	1	5				*OTU_99*	0	0	1	*OTU_942*	0	0	1	*OTU_1495*	0	1
OTU_131	0	3	2				*OTU_102*	1	0	0	*OTU_945*	0	1	0	*OTU_1559*	1	0
OTU_139	0	5	0				*OTU_107*	0	0	1	*OTU_954*	1	0	0	*OTU_1596*	1	0
OTU_179	5	0	0				*OTU_118*	0	1	0	*OTU_993*	0	1	0	*OTU_1614*	1	0
OTU_194	1	4	0				*OTU_133*	1	0	0	*OTU_995*	1	0	0	*OTU_1615*	0	1
OTU_367	4	1	0				*OTU_138*	1	0	0	*OTU_1008*	0	0	1	*OTU_1635*	0	1
OTU_502	3	2	0				*OTU_144*	0	0	1	*OTU_1025*	0	1	0	*OTU_1681*	0	1
OTU_520	1	4	0				*OTU_174*	0	1	0	*OTU_1098*	0	1	0	*OTU_1690*	0	1
OTU_597	1	3	1				*OTU_207*	0	0	1	*OTU_1107*	1	0	0	*OTU_1699*	1	0
OTU_646	3	1	1				*OTU_213*	1	0	0	*OTU_1109*	0	1	0	*OTU_1714*	0	1
OTU_737	1	3	1				*OTU_229*	1	0	0	*OTU_1155*	0	0	1	*OTU_1720*	1	0
OTU_790	0	5	0				*OTU_261*	1	0	0	*OTU_1165*	0	0	1	*OTU_1727*	1	0
OTU_846	0	0	5				*OTU_279*	0	0	1	*OTU_1193*	0	0	1	*OTU_1754*	0	1
OTU_886	0	0	5				*OTU_280*	0	0	1	*OTU_1200*	0	0	1	*OTU_1765*	1	0
OTU_908	1	4	0				*OTU_290*	1	0	0	*OTU_1218*	0	1	0	*OTU_1801*	1	0
OTU_1061	0	0	5				*OTU_303*	0	0	1	*OTU_1238*	0	0	1	*OTU_1843*	0	1
OTU_1062	0	1	4				*OTU_337*	0	0	1	*OTU_1260*	0	1	0			
OTU_1112	3	0	2				*OTU_346*	0	0	1	*OTU_1267*	0	1	0			
OTU_1117	0	4	1				*OTU_436*	1	0	0	*OTU_1309*	0	1	0			
OTU_1121	0	0	5				*OTU_455*	1	0	0	*OTU_1348*	1	0	0			
OTU_1156	0	0	5				*OTU_467*	0	1	0	*OTU_1349*	0	0	1			
OTU_1161	0	5	0				*OTU_472*	1	0	0	*OTU_1356*	1	0	0			
OTU_1177	0	5	0				*OTU_476*	0	0	1	*OTU_1379*	0	0	1			
OTU_1188	1	4	0				*OTU_485*	0	1	0	*OTU_1380*	1	0	0			
OTU_1205	0	5	0				*OTU_502*	1	0	0	*OTU_1415*	0	1	0			
OTU_1301	0	0	5				*OTU_520*	1	0	0	*OTU_1418*	0	0	1			
OTU_1327	0	0	5				*OTU_530*	0	1	0	*OTU_1449*	0	0	1			

OTU_1367	0	0	5				*OTU_550*	1	0	0	*OTU_1495*	0	0	1			
OTU_1746	1	0	4				*OTU_551*	0	1	0	*OTU_1500*	0	0	1			
OTU_1760	0	0	5				*OTU_596*	0	0	1	*OTU_1519*	0	1	0			
OTU_1809	0	4	1				*OTU_625*	0	0	1	*OTU_1521*	0	0	1			
OTU_1852	2	3	0				*OTU_679*	1	0	0	*OTU_1536*	0	1	0			
OTU_7	0	3	1				*OTU_747*	0	0	1	*OTU_1616*	0	0	1			
OTU_212	0	2	2				*OTU_766*	1	0	0	*OTU_1633*	0	0	1			
OTU_218	0	4	0				*OTU_777*	0	0	1	*OTU_1696*	0	0	1			
OTU_290	4	0	0				*OTU_781*	1	0	0	*OTU_1738*	0	0	1			
OTU_389	0	3	1				*OTU_782*	0	1	0	*OTU_1754*	0	0	1			
OTU_454	0	4	0				*OTU_785*	1	0	0	*OTU_1843*	0	0	1			
OTU_500	0	4	0				*OTU_797*	1	0	0	*OTU_1860*	0	0	1			
OTU_606	0	0	4				*OTU_851*	0	0	1	*OTU_1898*	0	0	1			
OTU_712	0	4	0				*OTU_882*	0	0	1	*OTU_1907*	0	0	1			
OTU_770	0	4	0				*OTU_919*	0	1	0							
OTU_793	2	2	0				*OTU_923*	1	0	0							
OTU_953	4	0	0				*OTU_993*	1	0	0							
OTU_1078	0	0	4				*OTU_1062*	0	1	0							
OTU_1132	0	0	4				*OTU_1077*	0	1	0							
OTU_1137	0	4	0				*OTU_1096*	0	1	0							
OTU_1189	0	0	4				*OTU_1174*	1	0	0							
OTU_1247	0	4	0				*OTU_1202*	1	0	0							
OTU_1266	0	0	4				*OTU_1221*	1	0	0							
OTU_1333	4	0	0				*OTU_1271*	0	1	0							
OTU_1364	0	0	4				*OTU_1279*	1	0	0							
OTU_1381	0	0	4				*OTU_1306*	1	0	0							
OTU_1473	0	0	4				*OTU_1310*	1	0	0							
OTU_1483	0	4	0				*OTU_1365*	0	1	0							
OTU_1583	0	0	4				*OTU_1392*	0	1	0							
OTU_1622	0	0	4				*OTU_1402*	0	1	0							

OTU_1683	2	2	0				*OTU_1410*	0	1	0							
OTU_1689	0	4	0				*OTU_1415*	0	1	0							
OTU_1715	0	0	4				*OTU_1426*	0	1	0							
OTU_1728	0	3	1				*OTU_1482*	0	1	0							
OTU_1745	0	0	4				*OTU_1497*	1	0	0							
OTU_1766	0	0	4				*OTU_1505*	1	0	0							
OTU_1781	0	4	0				*OTU_1624*	1	0	0							
OTU_1885	3	1	0				*OTU_1633*	1	0	0							
OTU_34	0	3	0				*OTU_1640*	0	0	1							
OTU_48	0	3	0				*OTU_1650*	0	0	1							
OTU_134	0	0	3				*OTU_1655*	0	1	0							
OTU_156	2	1	0				*OTU_1725*	0	0	1							
OTU_258	2	1	0				*OTU_1727*	1	0	0							
OTU_279	0	2	1				*OTU_1732*	1	0	0							
OTU_297	3	0	0				*OTU_1755*	1	0	0							
OTU_339	0	3	0				*OTU_1788*	0	1	0							
OTU_424	2	1	0				*OTU_1854*	0	0	1							
OTU_450	0	2	1				*OTU_1895*	0	0	1							
OTU_455	0	3	0				*OTU_1907*	1	0	0							
OTU_493	0	3	0				*OTU_1916*	0	1	0							
OTU_532	0	2	1														
OTU_648	1	2	0														
OTU_650	0	3	0														
OTU_709	0	3	0														
OTU_881	0	3	0														
OTU_889	0	3	0														
OTU_1123	0	3	0														
OTU_1143	0	0	3														
OTU_1150	0	2	1														
OTU_1310	3	0	0														

OTU_1337	1	2	0														
OTU_1354	0	0	3														
OTU_1361	0	0	3														
OTU_1394	1	1	1														
OTU_1500	0	2	1														
OTU_1523	0	3	0														
OTU_1533	0	3	0														
OTU_1534	0	3	0														
OTU_1565	1	2	0														
OTU_1586	3	0	0														
OTU_1588	0	3	0														
OTU_1595	0	3	0														
OTU_1611	0	3	0														
OTU_1620	0	3	0														
OTU_1666	0	3	0														
OTU_1673	0	0	3														
OTU_1680	0	0	3														
OTU_1707	0	1	2														
OTU_1721	0	3	0														
OTU_1722	1	2	0														
OTU_1729	0	0	3														
OTU_1753	0	0	3														
OTU_1825	0	0	3														
OTU_1837	0	3	0														
OTU_1856	0	3	0														
OTU_1900	0	0	3														
OTU_1903	0	0	3														
OTU_9	0	2	0														
OTU_16	0	2	0														
OTU_21	0	0	2														

OTU_45	0	2	0																
OTU_54	0	2	0																
OTU_56	0	2	0																
OTU_57	0	2	0																
OTU_60	2	0	0																
OTU_67	1	1	0																
OTU_71	2	0	0																
OTU_95	0	2	0																
OTU_97	0	2	0																
OTU_122	0	2	0																
OTU_170	0	2	0																
OTU_199	0	2	0																
OTU_247	0	2	0																
OTU_271	0	2	0																
OTU_277	1	1	0																
OTU_324	0	2	0																
OTU_329	0	1	1																
OTU_335	2	0	0																
OTU_342	0	2	0																
OTU_348	0	0	2																
OTU_355	2	0	0																
OTU_378	0	2	0																
OTU_382	0	2	0																
OTU_403	0	0	2																
OTU_416	0	1	1																
OTU_452	2	0	0																
OTU_485	0	1	1																
OTU_504	0	0	2																
OTU_604	0	2	0																
OTU_632	0	0	2																

OTU_667	2	0	0														
OTU_757	0	2	0														
OTU_788	1	1	0														
OTU_810	0	0	2														
OTU_900	2	0	0														
OTU_1056	0	2	0														
OTU_1096	0	2	0														
OTU_1099	0	2	0														
OTU_1200	0	2	0														
OTU_1262	2	0	0														
OTU_1270	2	0	0														
OTU_1280	0	2	0														
OTU_1386	0	2	0														
OTU_1388	0	2	0														
OTU_1430	0	1	1														
OTU_1482	0	1	1														
OTU_1624	0	1	1														
OTU_1665	0	2	0														
OTU_1732	0	1	1														
OTU_1807	0	2	0														
OTU_1863	0	2	0														
OTU_1886	1	1	0														
OTU_1895	1	1	0														
OTU_25	1	0	0														
OTU_31	1	0	0														
OTU_58	0	1	0														
OTU_75	0	0	1														
OTU_84	0	1	0														
OTU_89	0	1	0														
OTU_186	0	1	0														

OTU_203	0	1	0														
OTU_226	0	1	0														
OTU_230	0	1	0														
OTU_233	0	0	1														
OTU_240	0	0	1														
OTU_254	0	0	1														
OTU_272	0	1	0														
OTU_321	0	1	0														
OTU_336	0	1	0														
OTU_338	0	1	0														
OTU_407	0	1	0														
OTU_451	0	1	0														
OTU_475	0	1	0														
OTU_623	0	1	0														
OTU_637	0	1	0														
OTU_744	0	1	0														
OTU_805	0	0	1														
OTU_836	0	1	0														
OTU_872	0	1	0														
OTU_880	0	0	1														
OTU_914	0	1	0														
OTU_986	0	0	1														
OTU_989	0	1	0														
OTU_996	0	1	0														
OTU_1051	0	1	0														
OTU_1216	0	1	0														
OTU_1221	0	1	0														
OTU_1230	0	1	0														
OTU_1279	0	1	0														
OTU_1291	0	1	0														

OTU_1303	0	1	0														
OTU_1309	0	1	0														
OTU_1326	1	0	0														
OTU_1392	0	1	0														
OTU_1411	0	1	0														
OTU_1428	0	1	0														
OTU_1506	0	1	0														
OTU_1539	0	1	0														
OTU_1582	0	1	0														
OTU_1650	0	1	0														
OTU_1692	1	0	0														
OTU_1714	0	1	0														
OTU_1780	0	1	0														
OTU_1788	0	1	0														
OTU_1810	0	0	1														
OTU_1898	0	0	1														

Table S2. Comparison of rarefied (R) and non-rarefied (NR) data performance of the bacterial operational taxonomic units (OTUs) in all the investigated developmental stages of *Sparus aurata* larvae. Replicated samples: a, b, c.

	**Sequence reads**		**OTUs**		**S_{Chao} coverage**	
	NR	R	NR	R	NR	R
**D0a**	2487	335	130	45	0.972	0.934
**D0b**	4762	335	305	70	0.970	0.893
**D0c**	6327	335	217	70	0.970	0.884
**D5a**	6119	335	94	19	0.994	0.973
**D5b**	7626	335	91	12	0.991	0.988
**D15a**	4364	335	170	33	0.981	0.952
**D15b**	1321	335	127	36	0.987	0.949
**D15c**	2808	335	185	27	0.985	0.964
**D21a**	650	335	108	29	0.976	0.952
**D21b**	1539	335	150	36	0.982	0.949
**D21c**	1002	335	165	51	0.975	0.896
**D71a**	997	335	137	59	0.925	0.925
**D71b**	1696	335	144	57	0.969	0.937

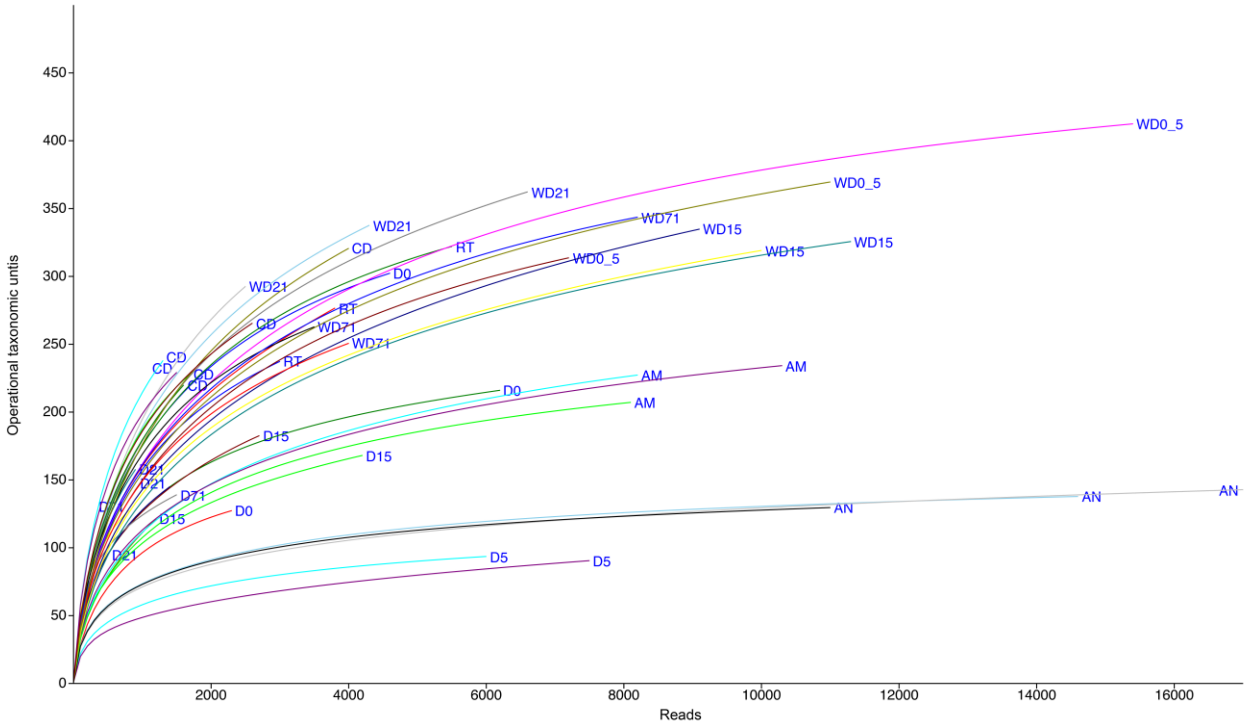

Figure S1. Rarefaction curves of the sequencing reads in all individual samples. Numbers indicate replicates. WD: Water sample on day X, D: days post hatching, CD: commercial diet, RT: rotifers, AN: Artemia nauplii, AM: Artemia metanauplii. Numbers indicate days post hatching

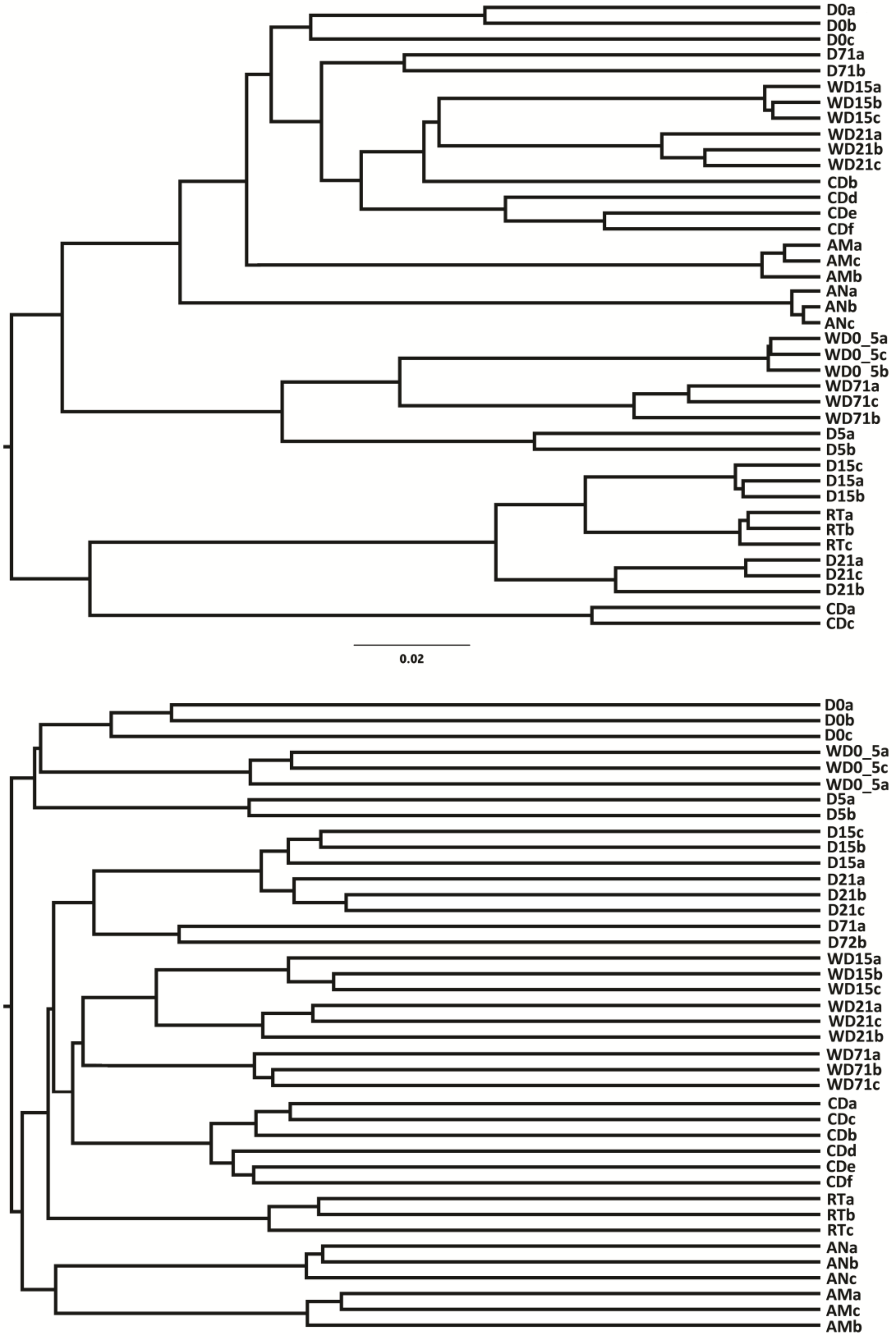

Figure S2. Clustering of bacterial communities at the operational taxonomic units level *Sparus aurata* larvae and the surrounding environment microbial populations. UPGMA clustering was performed on weighted (top) and unweighted (bottom) UNIFRAC measures between samples based on phylogenetic and OTU distribution distance matrices.

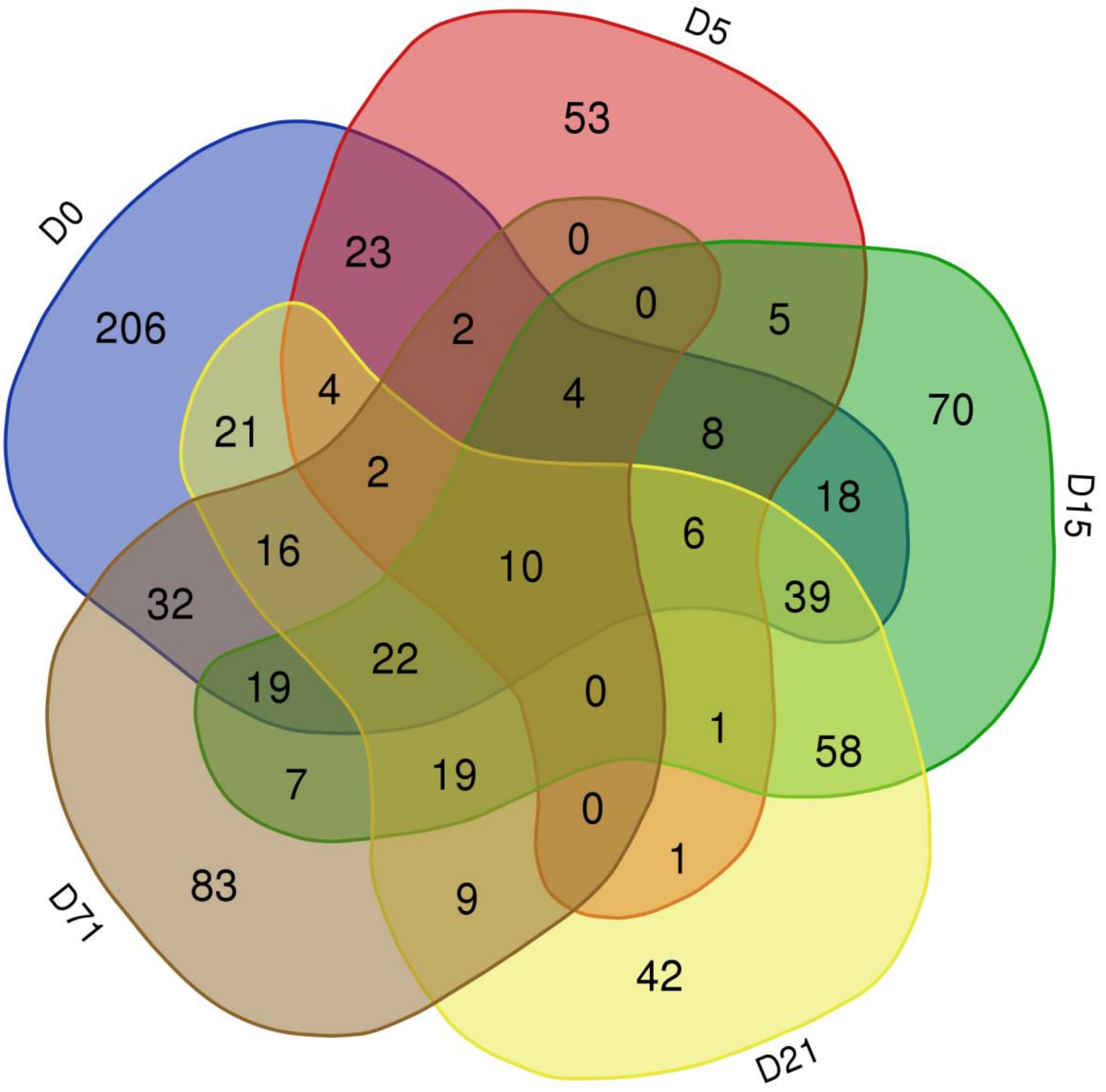

Figure S3. Venn diagram of the shared operational taxonomic units between all the investigated developmental stages of *Sparus aurata* larvae.

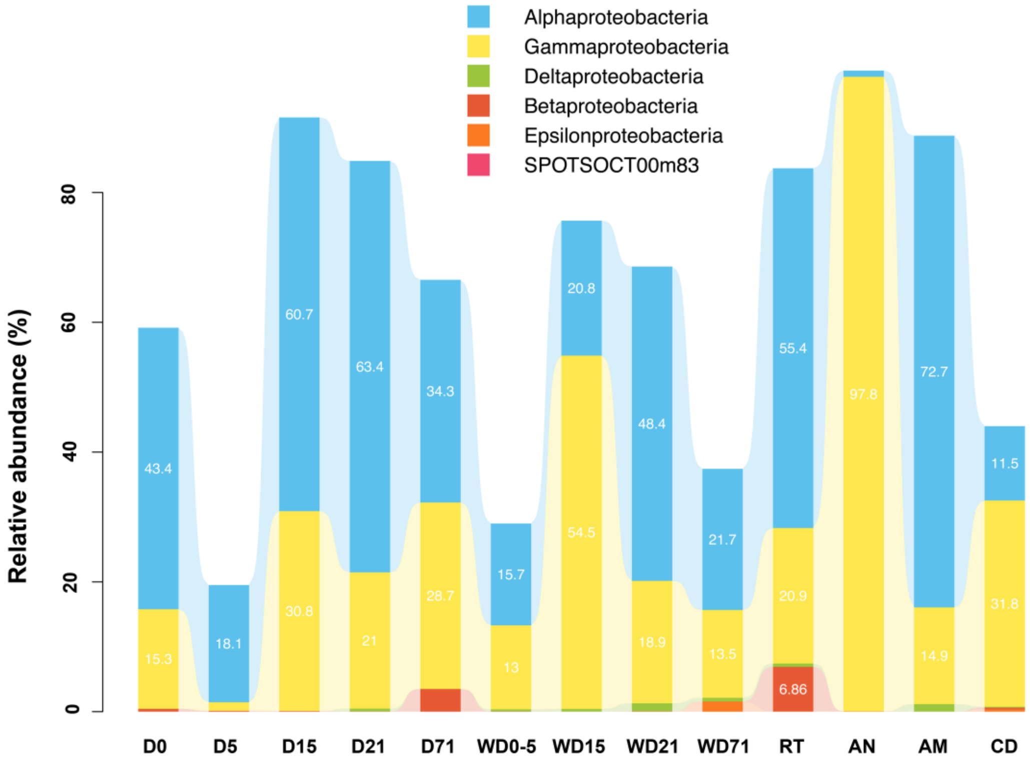

Figure S4. Mean relative abundance of proteobacterial classes in all sample categories (*Sparus aurata* larvae, rearing water and feeds) D: day, AM: *Artemia salina* metanauplii, AN: *Artemia salina* nauplii, CD: commercial diet, RT: rotifers, W: water sample.

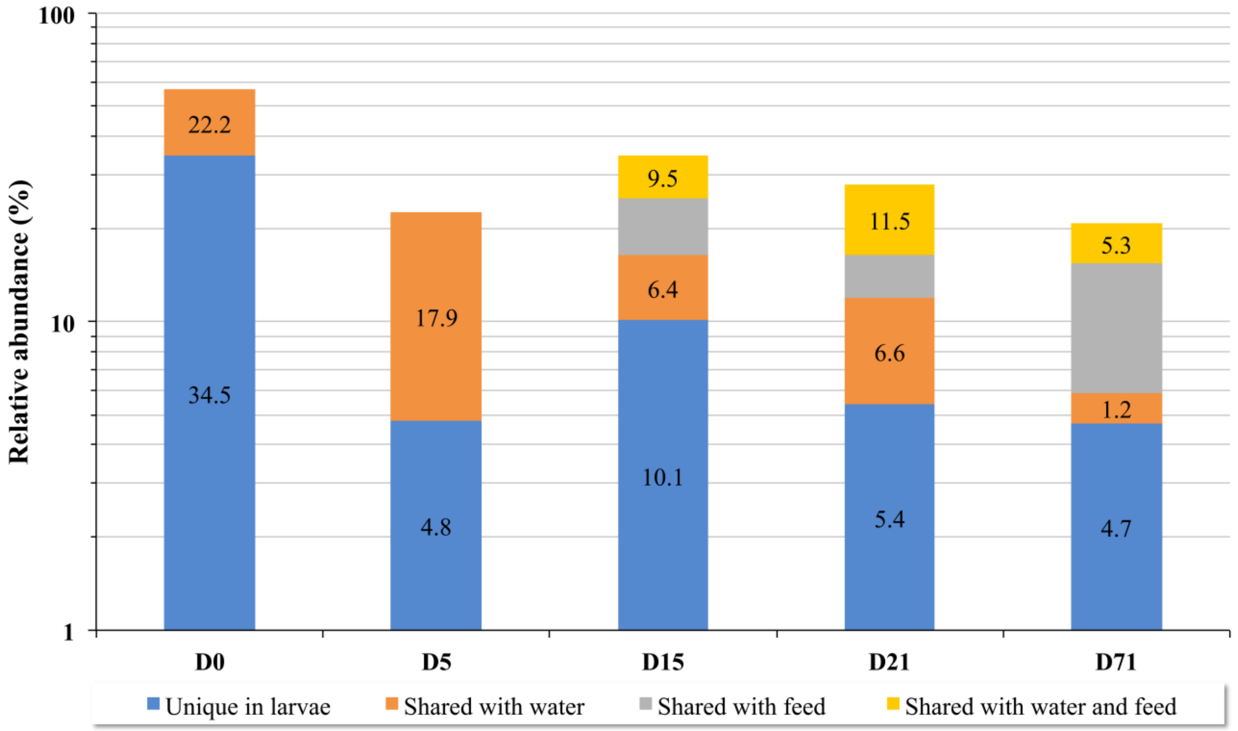

Figure S5. Percentage of shared operational taxonomic units between *Sparus aurata* larvae and the surrounding environment microbial populations. The percentage of unique OTUs in the water and feed is not shown.
